# A Conjugation Delivery System of Macrophages and Platelet Pharmacytes Promotes Regeneration After Spinal Cord Injury

**DOI:** 10.1002/advs.202513474

**Published:** 2025-12-25

**Authors:** Haoli Wang, Hao Hu, Yijun Li, Lintao Hu, Chenhui Gu, Yiwei Zhu, Jing Huang, Na Li, Shuqi Jiang, Shouyan Zu, Jiachen Xu, Yining Wang, Ke Yang, Pengfei Chen, Liqing Shangguan, Yongcheng Wang, Shunwu Fan, Xianfeng Lin, Qingqing Wang

**Affiliations:** ^1^ Department of Orthopaedic Surgery Sir Run Run Shaw Hospital Zhejiang University School of Medicine Hangzhou Zhejiang 310016 China; ^2^ Cixi Biomedical Research Institute Wenzhou Medical University Cixi Zhejiang 315302 China; ^3^ Key Laboratory of Mechanism Research and Precision Repair of Orthopaedic Trauma and Aging Diseases of Zhejiang Province Hangzhou Zhejiang 310016 China; ^4^ School of Basic Medical Sciences and Forensic Medicine Hangzhou Medical College Hangzhou Zhejiang 310013 China

**Keywords:** cellular combination delivery system, efferocytosis, gene delivery, mitochondria transfer, spinal cord injury

## Abstract

Mitochondrial dysfunction occurs in macrophages with efferocytosis defects, which hinders recovery from tissue injury. Targeting intercellular mitochondrial transfer is a promising therapy for augmenting cellular therapy. Here, this work elucidates the stress resistance capabilities of mitochondria in anucleate platelets and shows that platelets transfer mitochondria to macrophages under cellular stress, which restores impaired efferocytosis. This work devises a delivery system in which platelets are loaded with cationic polymers (NPs) for PPARγ overexpression and conjugated to macrophages (M‐P‐NPs@PPARγ). In this system, activated platelets induce mitochondrial transfer and release NPs into macrophages, increasing ATP production and maintaining lipid homeostasis. As a proof‐of‐concept, in representative efferocytosis‐deficit central nervous system disease spinal cord injury model, impaired efferocytosis is reversed by M‐P‐NP@PPARγ, resulting in neural regeneration and remyelination and ultimately promoting motor function recovery. In summary, this work has developed a strategy combining mitochondria and gene delivery to restore macrophage efferocytosis postinjury by regulating energy and lipid metabolism.

## Graphical abstract

A macrophage–platelet conjugation system (M‐P‐NPs@PPARγ) targets injure tissue to co‐deliver platelet mitochondria and PPARγ‐encoding NPs. This strategy boosts the macrophage energy supply and restores lipid homeostasis, leading to synergistically enhanced efferocytosis and tissue repair

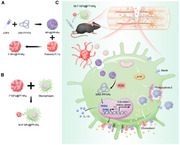

## 1 Introduction

Energy metabolism regulation is a crucial process during activation of the repair pathways of damaged tissues. Adequate adenosine triphosphate (ATP) generated through mitochondrial oxidative phosphorylation (OXPHOS) is essential for tissue repair.^[^ ^1^ ^]^ However, mitochondrial dysfunction, a common consequence of ischemic/hypoxic injury in diverse cells, underpins the pathophysiology of multiple disorders.^[^ ^2^ ^]^ This has positioned mitochondrial repair as a central strategy for treatment and tissue regeneration. Nevertheless, strategies including genetic induction of mitochondrial biogenesis^[^ ^3^ ^]^ and antioxidant protection of mitochondrial integrity,^[^ ^4^ ^]^ which tend to be ineffective if mitochondria are already dysfunctional. Recent studies have shown that cells are capable of acquiring mitochondria from other cell types to correct mitochondrial dysfunction.^[^ ^5^ ^]^ Therefore, transplanting exogenous mitochondria to restore mitochondrial homeostasis of dysfunctional cells is a promising strategy.^[^ ^6^ ^]^

As immune cells with high energy demands, macrophages are vital for tissue repair and regeneration through efferocytosis.^[^ ^7^ ^]^ Efferocytosis refers to the clearance of apoptotic cells by phagocytes to terminate the inflammatory response, activate pro‐tissue resolution pathways and prevent secondary necrosis.^[^ ^8^ ^]^ Mitochondrial OXPHOS serves as the primary energy source for efferocytosis, supporting the energy‐consuming process of actin cytoskeleton reorganization required for the engulfment and clearance of apoptotic cells.^[^ ^9^, ^10^ ^]^ Compared with proinflammatory macrophages, macrophages that rely mainly on OXPHOS usually polarize toward an anti‐inflammatory phenotype and are more efficient efferocytes.^[^ ^11^ ^]^ However, under damage conditions, mitochondrial dysfunction in macrophages leads to impaired OXPHOS, which, in turn, decreases efferocytosis.^[^ ^12^, ^13^ ^]^ Most efferocytosis‐deficit macrophages have a normal internalization ability but fail to appropriately digest apoptotic cells (FAD),^[^ ^14^ ^]^ which is mainly due to dysregulated intracellular lipid metabolism and energy deficit.^[^ ^15^, ^16^ ^]^ Thus, facilitating cellular lipid efflux and improving the mitochondrial OXPHOS of infiltrating macrophages to increase efferocytosis is critical for promoting tissue regeneration.

However, a critical barrier for exogenous mitochondria transplantation lies in identifying optimal donor cells, which must not only be recognized by recipient cells but also retain the capacity to initiate functional mitochondria delivery under stress conditions. Although cells such as adipocytes and cardiomyocytes are capable of transferring mitochondria to macrophages,^[^ ^17^, ^18^ ^]^ these mitochondria may be unable to improve macrophage function due to the high susceptibility of mitochondria following oxidative damage. In contrast, upon activation within an injured microenvironment, platelets release functional mitochondria, which are internalized by mesenchymal stromal cells and can significantly promote wound healing.^[^ ^19^ ^]^ Previous studies have shown that anucleate platelets have a very high ATP turnover rate, and this high energy demand is met primarily through glycolysis and mitochondrial OXPHOS.^[^ ^20^ ^]^ However, thrombosis and the rapid clearance of platelets within the tissue during the acute phase of injury limit the effective interaction between platelets and macrophages,^[^ ^21^, ^22^ ^]^ and the pathophysiological significance of mitochondrial transfer among non‐parenchymal cells in tissue repair and regeneration remains unclear. Therefore, identifying mitochondria that are highly resistant to oxidative stress and developing a therapeutic strategy that effectively promotes interactions between their donor cells and macrophages following tissue injury is urgently needed.

**Figure 1:**
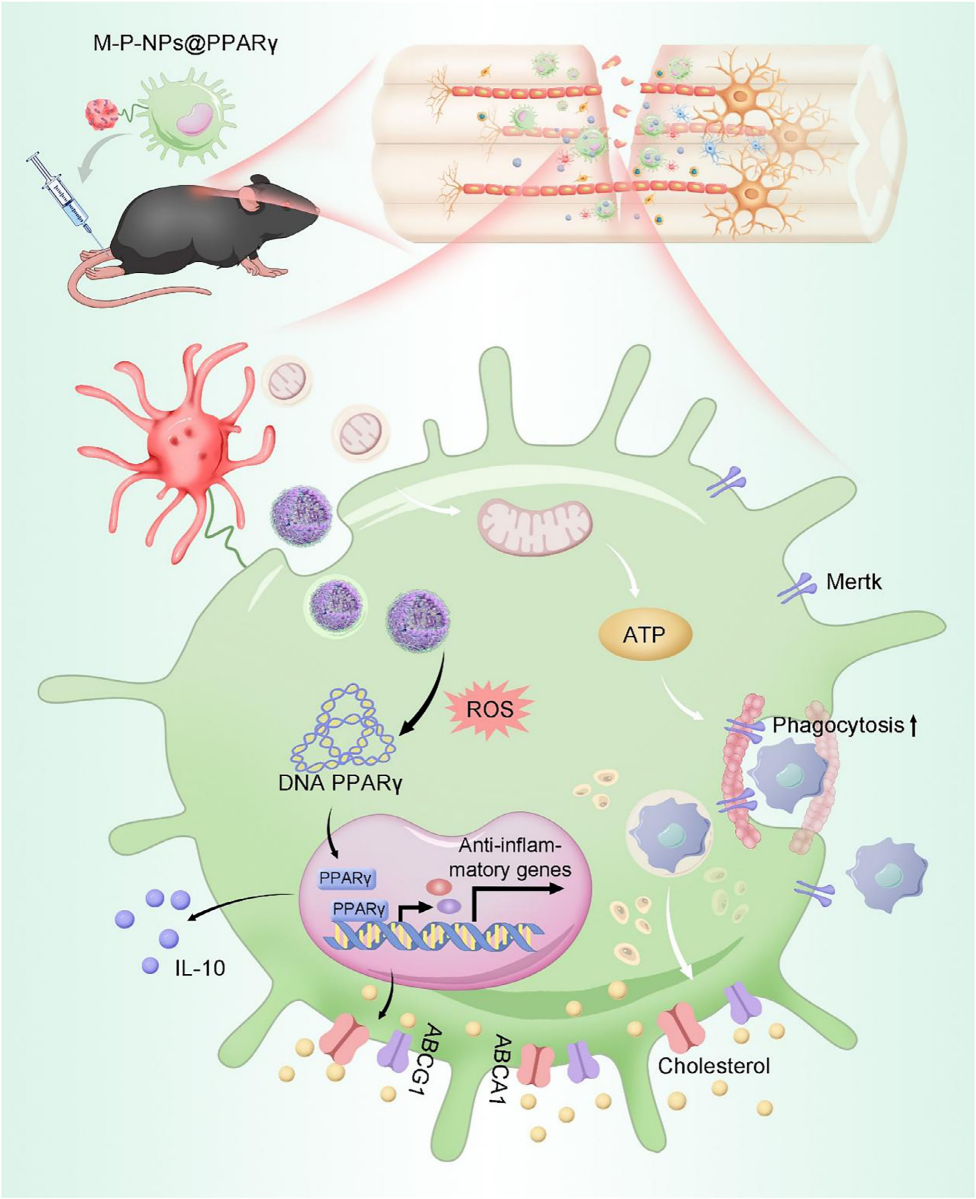
Schematic illustration of the therapeutic mechanism of M‐P‐NPs@PPARγ. The PPARγ overexpression plasmid was encapsulated with CBP5 to form NPs@PPARγ. Preparation of P‐NPs@PPARγ via encapsulation of NPs@PPARγ in the OCS of platelets, and the construction of M‐P‐NPs@PPARγ was mediated by conjugation of P‐NPs@PPARγ with the BMM plasma membrane through a click reaction. After intravenous delivery, M‐P‐NPs@PPARγ targeted disrupted tissue. Upon activation within an injured microenvironment, the platelets released NPs@PPARγ and functional mitochondria, which were internalized by macrophages. Transplanted platelet mitochondria increased ATP generation in macrophages, leading to actin polymerization and continual efferocytosis. The NPs@PPARγ entered the cytosol and released DNA in a ROS‐triggered manner, over‐expressing PPARγ in macrophages and maintaining lipid homeostasis. Overall, our therapeutic strategy involving M‐P‐NPs@PPARγ‐mediated targeted delivery of mitochondria and PPARγ synergistically enhanced efferocytosis and facilitated tissue repair.

Traumatic spinal cord injury (SCI) is a devastating neurological disorder leading to long‐term disability.^[^ ^23^ ^]^ In particular, one of the primary pathological mechanisms in SCI is the accumulation of foam cells, which results from impaired efferocytosis due to mitochondrial dysfunction in macrophages.^[^ ^24^ ^]^ In the present study, we found that platelet‐derived mitochondria conferred protection against oxidative damage and facilitated the repair of efferocytosis. Given that peroxisome proliferator‐activated receptor gamma (PPARγ) expression can regulate lipid metabolism and increase the expression of anti‐inflammatory genes, thereby augmenting efferocytosis,^[^ ^25^ ^]^ we combined our previous research on gene delivery systems^[^ ^26^ ^]^ and used a platelet open canalicular system (OCS) to load a reactive oxygen species (ROS)‐responsive cationic polymer (CBP5) for overexpressing PPARγ. To achieve targeted delivery, we applied a macrophage‒platelet combination delivery system to an SCI model to promote the transport of platelet‐derived pharmacytes to the injury site on the basis of the chemotaxis of macrophages.^[^ ^27^ ^]^ The conjugation of the macrophage plasma membrane with platelet pharmacytes was subsequently achieved through a click reaction.^[^ ^28^ ^]^ After intravenous injection, we found that the macrophage–platelet pharmacytes delivery system (designated M‐P‐NPs@PPARγ) effectively accumulated in the injured spinal cord and restored macrophage repair functions by releasing NPs to maintain lipid homeostasis and transferring functional mitochondria that can resist oxidative damage stress. Overall, we demonstrated that M‐P‐NPs@PPARγ significantly enhanced the efferocytosis of macrophages and exerted notable therapeutic effects against tissue damage (**Figure** **1**).

## 2 Results

### 2.1 Platelets Restore ATP Synthesis in Macrophages through the Transfer of Functional Mitochondria

**Figure 2:**
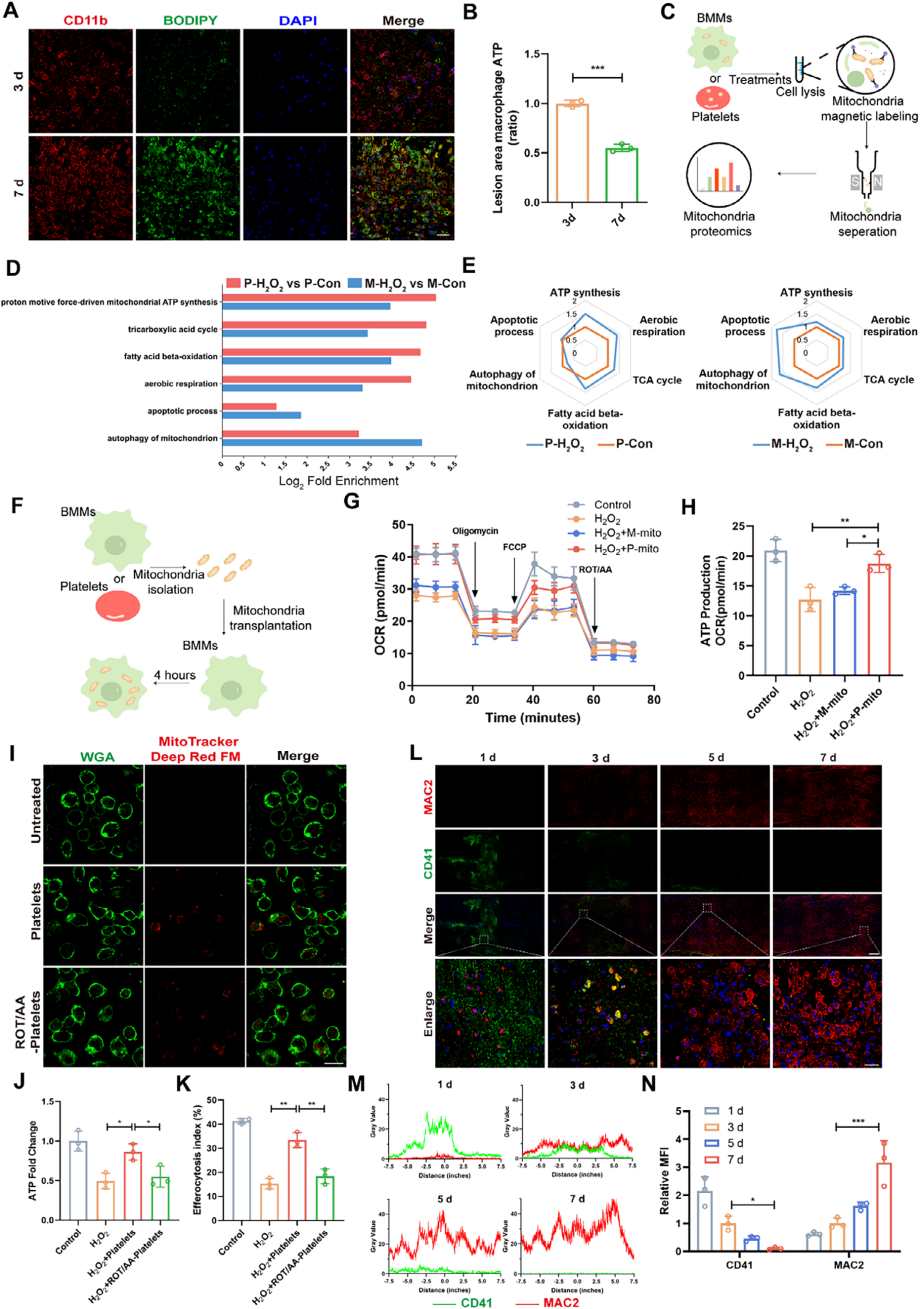
Effects of platelet‐derived mitochondria on the function of macrophages. A) The presence of lipid droplets (BODIPY, green) in macrophages (CD11b, red) at 3 and 7 days after SCI. Scale bar, 25 µm. B) ATP levels in sorted macrophages were determined (n = 3). C) Schematic showing the workflow of mitochondria isolation from BMMs and platelets. D) Significantly regulated proteins from the indicated conditions were used for GO biological process enrichment analysis. P‐H_2_O_2_, platelets treated with H_2_O_2_; P‐Con, platelets treated without H_2_O_2_; M‐H_2_O_2_, BMMs treated with H_2_O_2_; M‐Con, BMMs treated without H_2_O_2_; E) Radar plot illustrating the pathway enrichment score of ATP synthesis, aerobic respiration, the TCA cycle, fatty acid beta‐oxidation, the apoptotic process and autophagy of mitochondria in the P‐H_2_O_2_ group and the P‐Con group, the M‐H_2_O_2_ group and the M‐Con group. F) Diagram of the mitochondrial isolation and transplantation process. G) The OCRs of BMMs alone or treated with H_2_O_2_, H_2_O_2_ plus macrophage mitochondria or H_2_O_2_ plus platelet mitochondria were assessed using a Seahorse XF96 analyzer (n =3). M‐mito, macrophage mitochondria; P‐mito, platelet mitochondria; FCCP, carbonyl cyanide‐*p*‐trifluoromethoxyphenyl hydrazone; ROT/AA, rotenone and antimycin A. H) ATP production from Seahorse analysis (n = 3). I) Representative confocal microscopy images of BMMs (labeled with WGA‐FITC, green) after 12 h of incubation with platelets or platelets following exposure to ROT/AA and previous labeling with MitoTracker Deep Red FM (red). Scale bar, 20 µm. J) ATP levels in pathological macrophages were determined (n = 3). K) Quantitative analysis of efferocytosis in three independent experiments (n = 3). L–N) Immunofluorescence staining (L) and quantification (M and N) of CD41 (green) and MAC2 (red) in the SCI models (n = 3). Scale bar, 200 µm. Enlarged scale bar, 25 µm. All data are depicted as means ± SD; *p < 0.05, **p < 0.01, or ***p < 0.001.

Dysfunction of macrophages is an important factor underlying aberrant tissue repair.^[^ ^13^ ^]^ More lipid droplets were observed in macrophages on day 7 than on day 3 after SCI, and macrophages were similar to foam cells on day 7 after SCI (**Figure** **2**A), which could lead to a state of persistent injury.^[^ ^27^ ^]^ To investigate the cause of the dysfunction, ATP levels in macrophages (Ly6G^−^CD11c^−^CD11b^+^CD45^high^ cells) sorted from spinal cords via fluorescence‐activated cell sorting (FACS) were analyzed, revealing higher ATP levels on day 3 than on day 7, indicating that the mitochondrial energy status may be a significant factor influencing macrophage function (Figure 2B; Figure S1A, Supporting Information). Platelets can transfer functional mitochondria that reprogram the energy metabolism of neighboring cells.^[^ ^19^ ^]^ Gene Ontology (GO) analysis of the RNA sequencing (RNA‐Seq) dataset GSE206543 showed that compared to their precursor megakaryocytes, platelet‐enriched genes were mainly linked to ATP synthesis, lipid metabolic processes and the response to oxidative stress (Figure S1B, Supporting Information). The platelets presented upregulated expression of genes involved in ATP synthesis coupled with electron transport (ES = 0.26) (Figure S1C, Supporting Information). To further define the better ATP synthesis capacity of the mitochondria in platelets under oxidative damage stress, platelets and bone marrow‐derived macrophages (BMMs) were treated with or without H_2_O_2_, followed by mitochondrial immunoprecipitation and proteomics analysis (Figure 2C). Principal component analysis (PCA) revealed that the genes associated with M‐Con, M‐H_2_O_2_, P‐Con and P‐H_2_O_2_ were highly spatially distinct (Figure S1D, Supporting Information). Notably, we observed a predominant increase in inner mitochondrial membrane (IMM) proteins associated with OXPHOS in H_2_O_2_‐stimulated platelets, whereas macrophages did not exhibit such pronounced alterations (Figure S1E–G, Supporting Information). GO enrichment analysis confirmed that platelet mitochondria can protect against oxidative stress‐induced damage (Figure 2D). In general, ATP synthesis, aerobic respiration, the TCA cycle and fatty acid beta‐oxidation were increased, and the apoptotic process and autophagy of mitochondria in platelets treated with H_2_O_2_ were decreased, which contrasts with the findings in BMMs (Figure 2E). To investigate the metabolic status of platelet or macrophage mitochondria‐treated BMMs (Figure 2F), oxygen concentration rate (OCR) measurements were performed, which revealed impaired respiration in cells after H_2_O_2_ stimulation. Compared with treatment with macrophage mitochondria, supplementation with platelet mitochondria significantly increased respiration in BMMs (Figure 2G,H). This finding suggests that platelet‐derived mitochondria possess superior anti‐stress capabilities and can restore the energy status of BMMs within an injury microenvironment.

Next, to identify whether mitochondria transfer from platelets to macrophages occurred, we cocultured BMMs with platelets for 12 h. Representative confocal images visualized the presence of MitoTracker Deep Red FM‐labeled platelet mitochondria inside WGA‐FITC‐labeled BMMs, confirming the transfer of functional mitochondria from platelets to macrophages.  Furthermore, ROT/AA treatment did not affect their uptake efficiency (Figure 2I). Consistent with the OCR results, coculture of BMMs with platelets increased their ATP content compared with that of untreated platelets, while coculture with ROT/AA‐treated platelets attenuated this effect (Figure 2J). To further determine whether platelets transfer mitochondria to BMMs in vivo, we directly injected mitochondria‐labeled platelets into the injured region 1 day after SCI, and the transplanted platelet mitochondria were observed in the macrophages (Figure S2A,B, Supporting Information). We next determined whether the transport of mitochondria from platelets to BMMs regulates their efferocytosis activity. A classic apoptotic cell model of efferocytosis was established by co‐incubating BMMs and apoptotic Jurkat cells.^[^ ^11^ ^]^ When BMMs were subjected to H_2_O_2_, BMMs cultured with platelets phagocytosed more apoptotic cells (ACs) than untreated platelets did (Figure 2K; Figure S2C, Supporting Information). In contrast, platelets treated with ROT/AA failed to promote efferocytosis in BMMs. Collectively, these data suggest that platelets can transfer functional mitochondria to macrophages under oxidative damage stress and that this process improves the efferocytosis and therapeutic efficacy of macrophages.

Different types of cells infiltrate the injury site in sequential phases during tissue repair. Immunofluorescence (IF) assays showed that the macrophage infiltration in the injury sites of SCI was low on day 1, but increased on day 3 and peaked on day 7 (Figure 2L–N). Conversely, since platelets immediately participate in the first stage of wound healing after vascular damage to stop hemorrhages, they accumulated immediately after injury, decreased after day 1 and were absent on day 7. Similar infiltration characteristics were also demonstrated in a femoral defect model (Figure S3A–C, Supporting Information). These results suggest that macrophages can only interact with platelets at the injury site for a brief period (Figure S3D, Supporting Information).

### 2.2 Preparation and Characterization of P‐NPs@PPARγ

**Figure 3:**
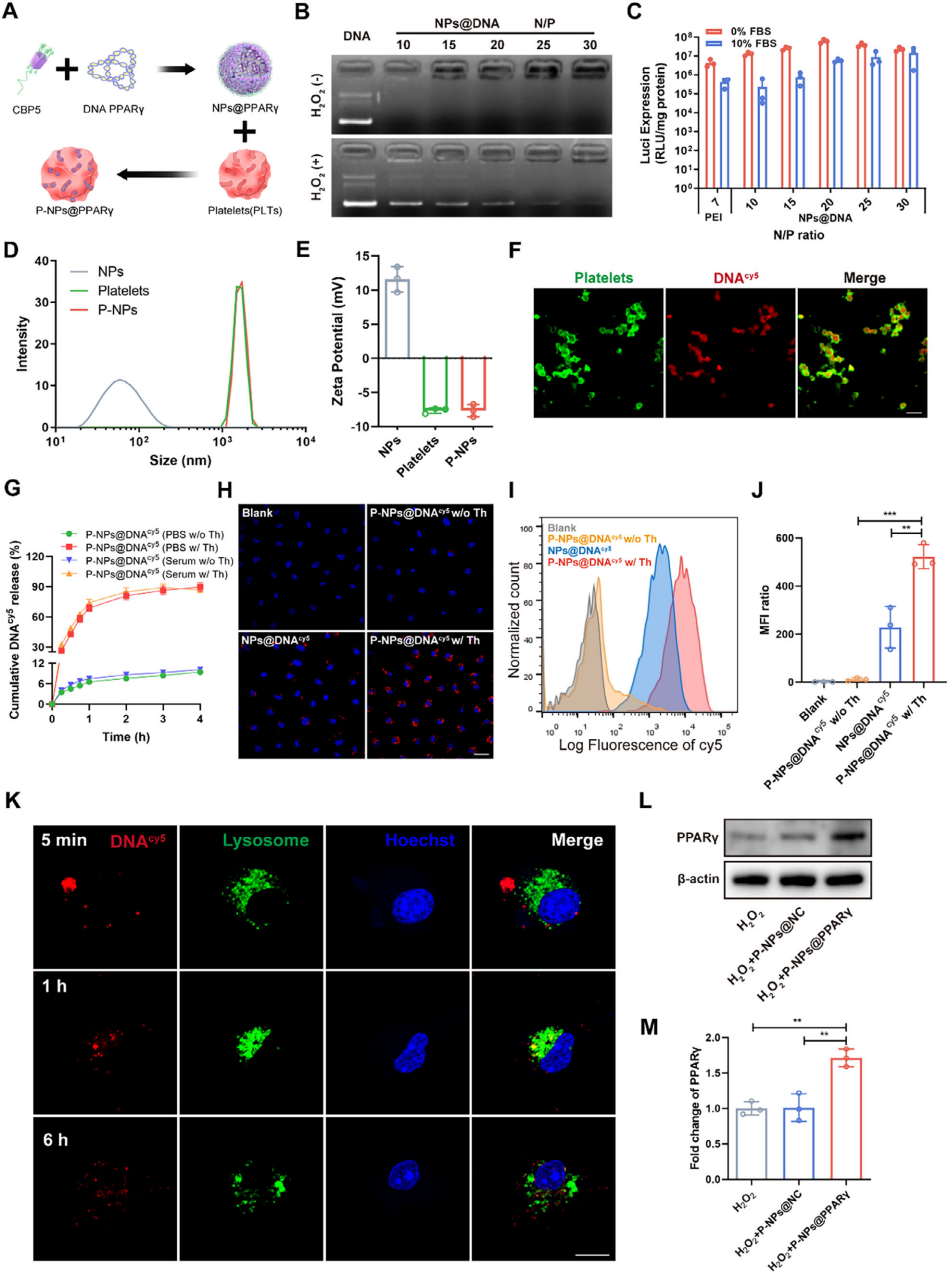
Characterization and cellular uptake of P‐NPs@DNA in BMMs. A) Schematic of plasmid DNA loading into CBP5 (NPs@DNA) with encapsulation by the OCS of platelets (P‐NPs@DNA). B) Gel retardation assay of NPs at different N/P ratios after a 1 h incubation at 37 °C with or without H_2_O_2_. C) Luciferase expression in RAW264.7 cells treated with NPs at different N/P ratios with or without 10% serum‐containing medium (n = 3). D) Size distribution analysis of the NPs, platelets and P‐NPs. E) The surface zeta potential of the NPs, platelets and P‐NPs. F) Representative confocal microscopy image of P^FITC^‐NPs@DNA^Cy5^. The platelet membrane was stained with WGA‐FITC (green), and the DNA was stained red. Scale bar, 5 µm. G) Cumulative DNA^Cy5^ release from P‐NPs@DNA^Cy5^ in PBS and serum‐containing medium, with or without 0.5 U mL^−1^ Th treatment. H) Representative fluorescence visualization showing the uptake of DNA by BMMs. DNA, red; Hoechst, blue. Scale bars, 25 µm. I) Cy5‐positive BMMs were measured by flow cytometry after treatment with NPs@DNA^Cy5^ or P‐NPs@DNA^Cy5^ with or without 0.5 U mL^−1^ Th treatment. J) Mean fluorescence intensity (MFI) of Cy5 (n = 3). K) Fluorescence visualization of DNA^Cy5^ and lysosome localization in BMMs after incubation with P‐NPs@DNA^Cy5^ for 5 min, 1 h and 6 h. Scale bar, 10 µm. DNA, red; nuclei, blue; lysosomes, green. L,M) PPARγ protein levels in BMMs (n = 3). All data are depicted as means ± SD; **p < 0.01, or ***p < 0.001.

A previous study demonstrated that the upregulation of PPARγ expression reduced foam macrophage formation and promoted efferocytosis.^[^ ^25^ ^]^ We therefore aimed to efficiently overexpress PPARγ in macrophages post‐injury. Since reactive oxygen species (ROS) levels in macrophages are increased after injury,^[^ ^29^ ^]^ we adjusted the previously designed ROS‐triggered cationic polymer CBP5 and complexed it with plasmid PPARγ DNA to generate NPs, which were encapsulated into platelet OCSs for effective delivery (**Figure** **3**A). As detected by gel electrophoresis, the release of DNA by NPs@DNA was significantly promoted by H_2_O_2_, confirming their ROS‐responsive release ability (Figure 3B). The overall gene transfection efficiency of CBP5 was then assessed using luciferase expressing‐plasmids as the reporter genes in RAW 264.7 cells. NPs with an *N/P* ratio of 20 achieved the highest transfection efficiency in serum‐free medium, which was nearly 10 times greater than PEI with an *N/P* of 7 (Figure 3C), suggesting that CBP5 can facilitate efficient gene transfection. At the optimal *N/P* ratio of 20, spherical NPs with an average diameter of 60.0 nm and a zeta potential of +11.6 mV were synthesized (Figure 3D,E; Figure S4A, Supporting Information). The size of the platelet‐coated NPs (P‐NPs) was slightly larger, while their zeta potential (−7.63 mV) was close to that of the platelets, indicating the successful loading of the NPs into the platelets (Figure 3D,E). To further verify the loading of the NPs, the P‐NPs were observed via confocal fluorescence microscopy. As shown in Figure 3F, red fluorescence (plasmid DNA labeled with Cy5, DNA^cy5^) was distributed inside the WGA‐FITC‐labeled platelets. Moreover, platelet function was preserved, as observed by filopodium formation after activation by scanning electron microscopy (SEM) (Figure S4B, Supporting Information). Furthermore, thrombin (Th) activation triggered a rapid release in the first hour and led to the almost complete releasing within 4 h, in both PBS and serum‐containing medium.^[^ ^30^ ^]^ In contrast, less than 10% of the DNA^cy5^ was released from P‐NPs@DNA^cy5^ without activation, confirming the stability of the P‐NPs (Figure 3G). These results indicated that platelets remained inactive during NPs encapsulation, whereas thrombin exposure activated P‐NPs@DNA.

To elucidate the function of P‐NPs, we first investigated the expression of proteins with or without thrombin activation. The preservation of key proteins after activation suggested that platelet function was preserved (Figure S4C, Supporting Information). Then, the interaction between P‐NPs@DNA^cy5^ and BMMs was assessed. As shown in Figure 3H, DNA^cy5^ was efficiently internalized by the BMMs after 4 h of incubation with thrombin. In contrast, P‐NPs@DNA^cy5^ without thrombin treatment showed a markedly lower uptake level. Moreover, P‐NPs@DNA^cy5^ exhibited a greater level of internalization than NPs@DNA^cy5^ in 10% serum medium. The results indicated that P‐NPs can improve the serum stability of the NPs and can be activated in the injury microenvironment to release the NPs and promote their transport into the BMMs (Figure 3H–J). Furthermore, DNA^cy5^ (red) with NPs (*N/P* ratio of 20) was internalized within only 5 min after activation. Red fluorescence was colocalized with LysoTracker Green‐labeled lysosomes at 1 h, while little red fluorescence was associated with lysosomes, and some red fluorescence was found in the nuclei at 6 h (Figure 3K), suggesting that the NPs efficiently escaped from lysosomes after cellular internalization. We next determined whether PPARγ could be successfully overexpressed by P‐NPs@PPARγ in BMMs. The real‐time quantitative polymerase chain reaction (RT‒qPCR) and WB results revealed that P‐NPs@NC exhibited negligible effects on PPARγ mRNA and protein levels after H_2_O_2_ stimulation. In comparison, BMMs treated with P‐NPs@PPARγ presented significantly higher PPARγ levels (Figure 3L,M; Figure S4D, Supporting Information). These results suggest that the P‐NPs upregulated PPARγ expression under injury conditions.

### 2.3 P‐NPs@PPARγ Improve Efferocytosis and Facilitate the Engulfment of Myelin Debris

**Figure 4:**
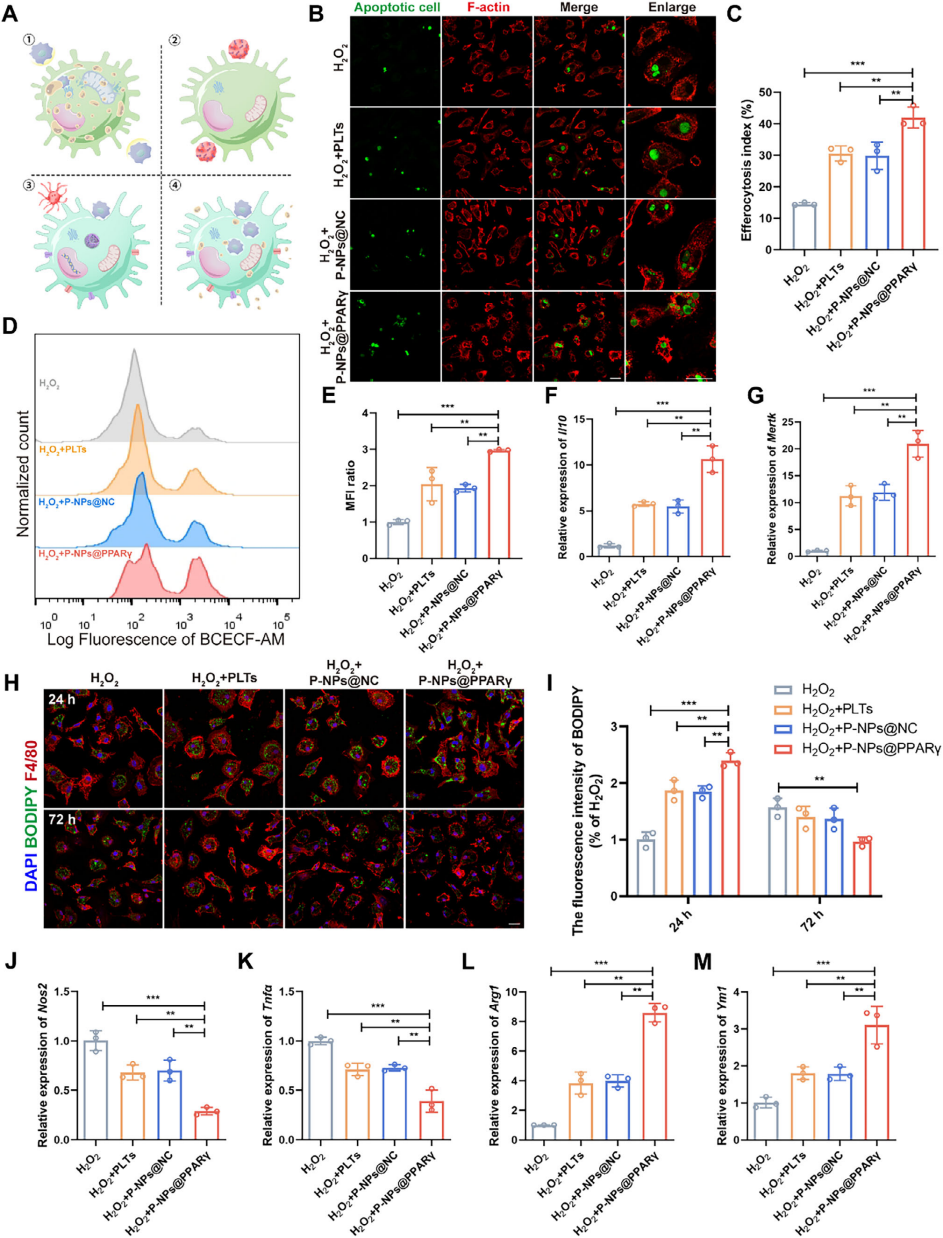
P‐NPs@PPARγ enhance efferocytosis and engulfment of myelin debris in vitro. A) Schematic of the restoration of defective efferocytosis by P‐NPs@PPARγ. B) Representative confocal microscopy analysis to evaluate efferocytosis by BMMs in vitro. Apoptotic Jurkat cells are in green; BMMs are in red. Scale bar, 25 µm. C) Quantitative analysis of efferocytosis in three independent experiments (n = 3). D) AC‐positive BMMs determined by flow cytometry after treatment. E) MFI of BCECF‐AM (n = 3). F, G) mRNA expression of MerTK and IL‐10 in BMMs under different treatment conditions upon stimulation with 200 µM H_2_O_2_ (n = 3). H) BMMs were incubated with myelin debris for the indicated times under different treatment conditions upon stimulation with 200 µM H_2_O_2_ and stained with F4/80 (red) and BODIPY (green). I) Quantification of the fluorescence intensity (BODIPY level) from H. J–M) mRNA expression of *Nos2*, *Tnfα*, *Arg1* and *Ym1* in BMMs engulfing myelin debris under different treatment conditions upon stimulation with 200 µM H_2_O_2_ (n = 3). All data are depicted as means ± SD; **p < 0.01, or ***p < 0.001.

We previously demonstrated that the therapeutic effects of platelets rely on mitochondrial transportation, and we hypothesized that P‐NPs@PPARγ combined with the regulation of lipid metabolism and promotion of ATP generation could effectively correct impaired efferocytosis (**Figure** **4**A). To investigate this hypothesis, we exposed BMMs to P‐NPs@PPARγ prior to stimulation with H_2_O_2_. BMMs treated with either platelets or P‐NPs@NC displayed improved phagocytosis of ACs, indicating that an increase in ATP levels enhanced efferocytosis, while BMMs cultured with P‐NPs@PPARγ engulfed more ACs than those subjected to the other treatments did (Figure 4B,C). The results of flow cytometric analysis also suggested that, compared with other treatments, P‐NPs@PPARγ enhanced the uptake of BCECF‐AM‐labeled ACs (Figure 4D,E). Compared with other treatments, P‐NPs@PPARγ significantly upregulated the expression of MerTK and IL‐10, which are important factors in efferocytosis,^[^ ^8^, ^31^ ^]^ further supporting the pro‐efferocytotic function of P‐NPs@PPARγ (Figure 4F,G). To evaluate the regulation of lipid homeostasis by P‐NPs@PPARγ, we next investigated the kinetics of myelin debris engulfment by BMMs. After 24 h of incubation with myelin debris, treatment of BMMs with P‐NPs@PPARγ resulted in greater accumulation of intracellular lipids, suggesting stronger myelin debris clearance activity. Moreover, the administration of P‐NPs@PPARγ quickly removed myelin‐induced lipids from BMMs within 72 h, indicating BMMs treated with P‐NPs@PPARγ had a strong ability to process accumulated lipids and maintain lipid homeostasis (Figure 4H,I). In addition, these treatments decreased the production of proinflammatory cytokines (*Nos2* and *Tnfα*) and increased the production of anti‐inflammatory cytokines (*Arg1* and *Ym1)* after 72 h of incubation with myelin debris, and the P‐NPs@PPARγ group outperformed the other groups (Figure 4J–M). Collectively, our results demonstrate that the regulation of lipid metabolism by P‐NPs@PPARγ could synergistically lead to the transition of macrophages toward an anti‐inflammatory phenotype.

### 2.4 RNA‐Seq Confirms P‐NPs@PPARγ Treatment Enhanced Efferocytosis

**Figure 5:**
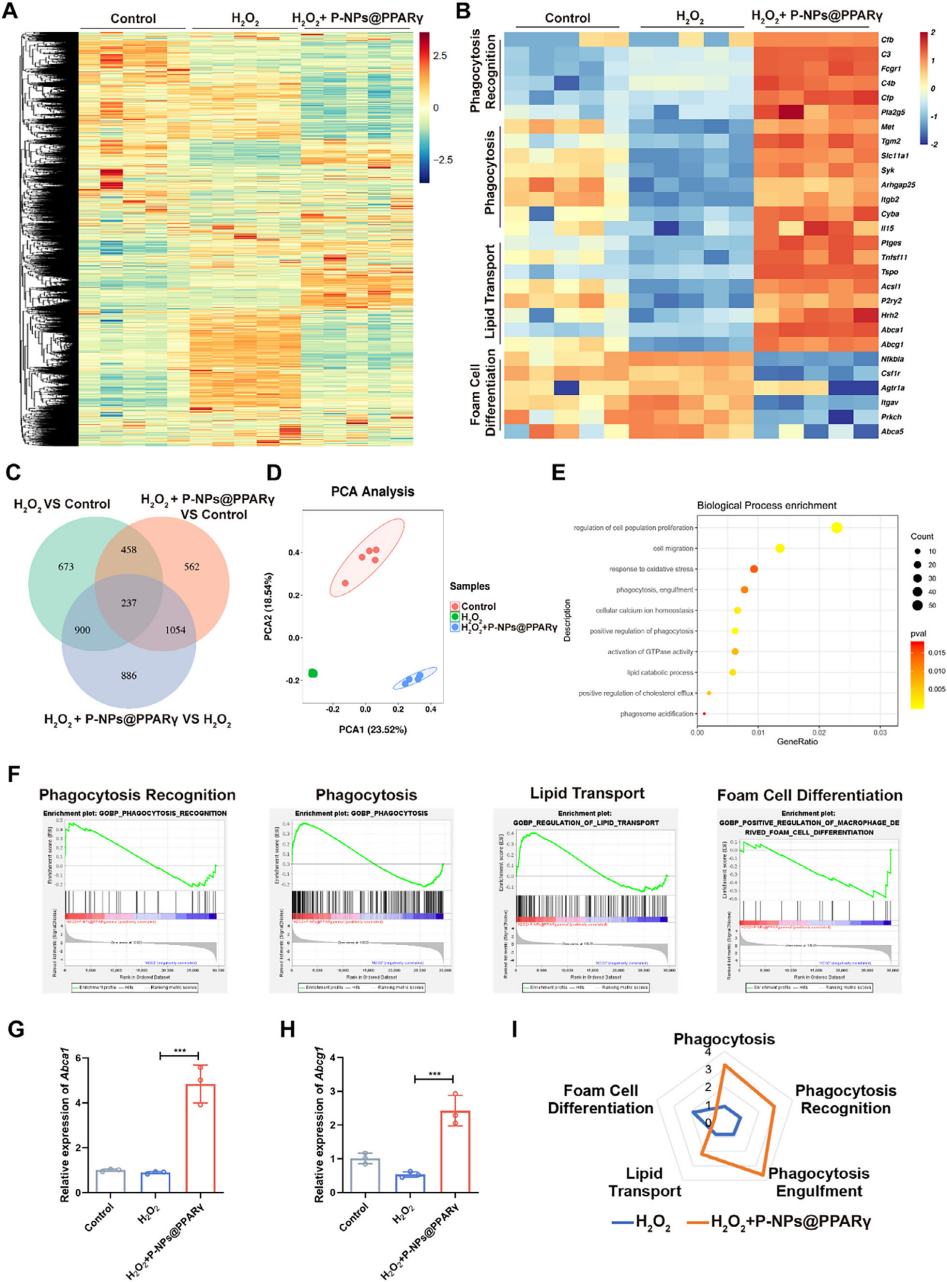
RNA‐Seq verification of the promotion of efferocytosis by P‐NPs@PPARγ. A) Heatmap showing differentially expressed genes in BMMs. BMMs were treated with H_2_O_2_ or H_2_O_2_+P‐NPs@PPARγ (n =5). B) Heatmap showing differentially expressed genes in macrophages. Macrophages were treated with H_2_O_2_ or H_2_O_2_+P‐NPs@PPARγ; n = 5 in different groups. C) Venn diagram of differentially expressed genes (absolute log2‐fold change ≥ 1; *p* < 0.05) detected in macrophages after treatment with H_2_O_2_ or H_2_O_2_+P‐NPs@PPARγ. D) PCA of genes in macrophages after treatment with H_2_O_2_ or H_2_O_2_+P‐NPs@PPARγ. E) Gene Ontology (GO) enrichment analysis of genes differentially expressed between the H_2_O_2_ and H_2_O_2_+P‐NPs@PPARγ groups. F) GSEA was applied to compare the gene sets involved in phagocytosis recognition, phagocytosis, lipid transport, and foam cell differentiation between the H_2_O_2_ and H_2_O_2_+P‐NPs@PPARγ groups. G, H) Relative *Abca1* and *Abcg1* mRNA levels in different groups (n = 3). I) Radar plot illustrating the pathway enrichment scores of the phagocytosis, phagocytosis recognition, phagocytosis engulfment, lipid transport, and foam cell differentiation pathways in the H_2_O_2_ and H_2_O_2_+P‐NPs@PPARγ groups. All data are depicted as means ± SD; ***p < 0.001.

To investigate the transcriptional changes in BMMs after P‐NPs@PPARγ treatment, we treated mouse BMMs as described above, followed by incubation with apoptotic human Jurkat cells, which enabled us to distinguish the expression of genes in a cell type‐specific manner after RNA‐Seq. Combined transcriptomic analysis was performed on H_2_O_2_‐stimulated BMMs with or without P‐NPs@PPARγ treatment. In total, 29 658 genes and 4770 differentially expressed genes among the three groups were identified (**Figure** **5**A–C). PCA revealed that the genes of the control, H_2_O_2_, and H_2_O_2_+P‐NPs@PPARγ groups were highly spatially distinct (Figure 5D). H_2_O_2_‐induced ROS production leads to decreased efferocytosis.^[^ ^32^ ^]^ This finding is consistent with our Gene Ontology (GO) analysis, which revealed that the differentially expressed genes were enriched mainly in the efferocytosis process (Figure 5E). We next used gene set enrichment analysis (GSEA) to compare the genes between the H_2_O_2_ and H_2_O_2_+P‐NPs@PPARγ groups. The H_2_O_2_+P‐NPs@PPARγ group showed upregulated expression of genes involved in phagocytosis recognition (ES = 0.47), phagocytosis (ES = 0.41), and lipid transport (ES = 0.41) and downregulated expression of genes involved in foam cell differentiation‐related genes (ES = −0.58) (Figure 5F). ABCA1 and ABCG1 induced by PPARγ activation are two major lipid transporters that improve lipid efflux and reduce foam cell formation.^[^ ^33^ ^]^ We further validated the changes in ABCA1 and ABCG1 expression in BMMs. The RT‒qPCR results revealed increased ABCA1 and ABCG1 transcript levels in the H_2_O_2_+P‐NPs@PPARγ group (Figure 5G,H). Overall, a radar chart (Figure 5I) showed enhanced efferocytosis function and diminished foam cell differentiation associated with P‐NPs@PPARγ treatment.

### 2.5 Distribution of M‐P‐NPs@PPARγ in SCI

**Figure 6:**
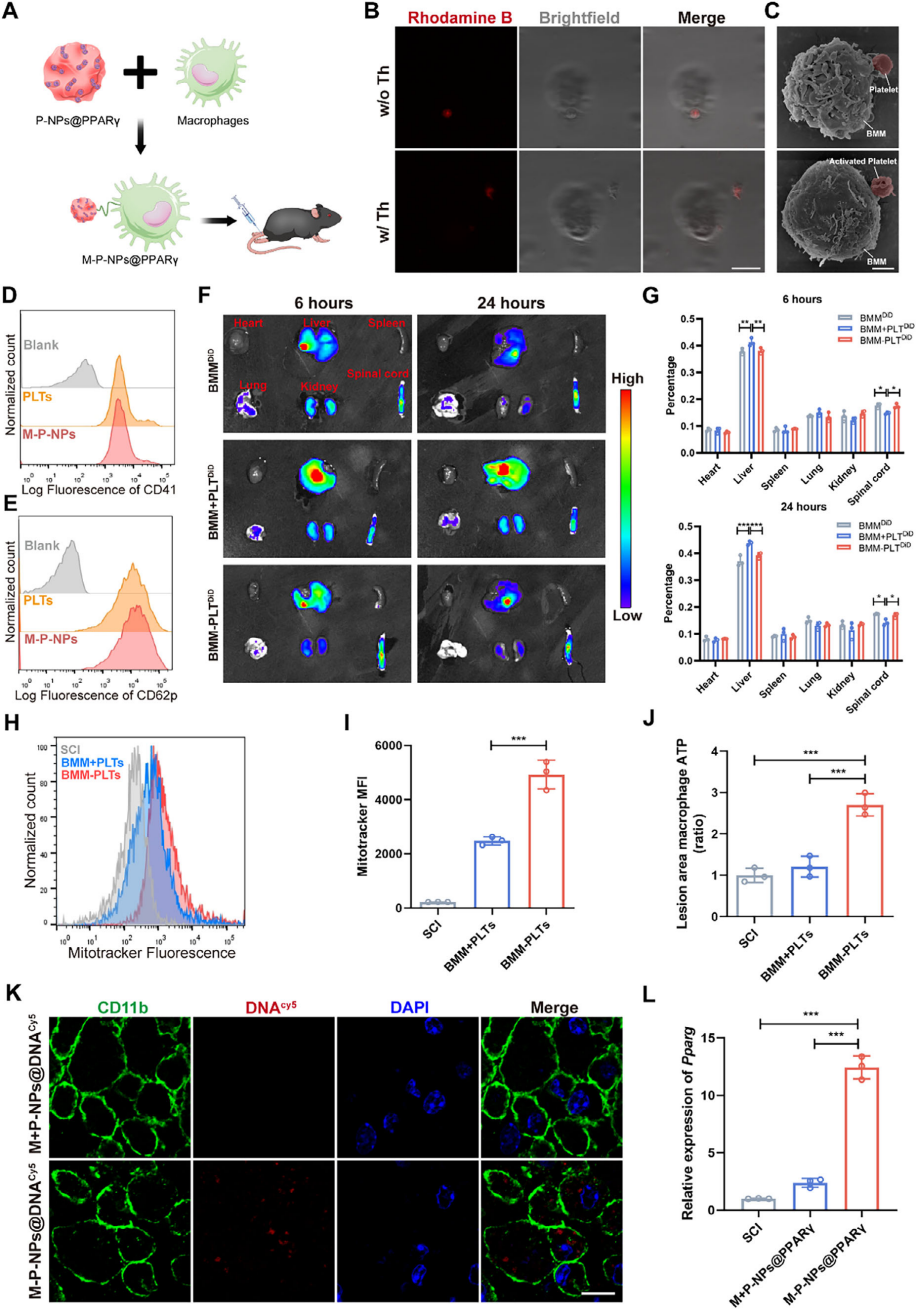
Targeting of M‐P‐NPs@PPARγ. A) Schematic illustration of M‐P‐NPs@PPARγ treatment in mice after SCI. B) Representative confocal microscopy images of M‐P‐NPs@PPARγ (platelets labeled with rhodamine B, red). Scale bar, 10 µm. C) SEM images of M‐P‐NPs@PPARγ. Scale bar, 2 µm. D) Flow cytometry analysis of CD41 expression in native platelets and M‐P‐NPs. E) CD62p was detected after platelet activation. F,G) Representative images of the biodistribution of different treatments in the heart, liver, spleen, lung, kidneys, and spinal cord 6 and 24 h after intravenous administration of BMM^DiD^, BMM+PLT^DiD^ or BMM‐PLT^DiD^. H) Representative flow cytometry histogram of the internalization of MitoTracker Deep Red‐labeled mitochondria from platelets in the lesion area. I) Flow cytometry quantification of the transfer of mitochondria from MitoTracker Deep Red‐labeled platelets to macrophages 24 h after injection (n = 3). J) ATP levels in sorted macrophages from different groups were determined (n = 3). K) Fluorescence images of spinal cord sections stained with an anti‐CD11b antibody after treatment with M+P‐NPs@DNA^Cy5^ or M‐P‐NPs@DNA^Cy5^. Scale bars, 10 µm. CD11b, green; nuclei, blue; DNA^Cy5^, red. L) Relative *Pparg* mRNA levels in different groups (n = 3). All data are depicted as means ± SD; *p < 0.05, **p < 0.01, or ***p < 0.001.

The development of functionalized cell therapies has been increasingly recognized as a promising strategy in regenerative medicine.^[^ ^34^ ^]^ Given that many macrophages can infiltrate lesion sites via various adhesion molecules and chemokine gradients after tissue injury,^[^ ^27^ ^]^ and the infiltration of platelets was decreased as shown above, we developed a macrophage–platelet pharmacytes delivery system (M‐P‐NPs@PPARγ) to transport P‐NPs@PPARγ into the injured spinal cord (**Figure** **6**A). Successful conjugation between macrophages and P‐NPs (M‐P‐NPs) was subsequently demonstrated via confocal fluorescence microscopy and SEM. The morphologies of the BMMs and platelets were maintained after conjugation.^[^ ^28^ ^]^ Additionally, platelet function was preserved, as evidenced by the preservation of filopodia shown by SEM and the expression of key proteins confirmed by flow cytometry after activation (Figure 6B–E). Moreover, platelet did not significantly affect macrophage viability or M1/M2 polarization, indicating platelets have little impact on macrophage function without activation (Figure S5A–E, Supporting Information). We further explored the injury lesion‐targeting capacity of M‐P‐NPs@PPARγ. BMMs and platelets were labeled with DiD (designated BMM^DiD^ and PLT^DiD^, respectively). BMM^DiD^, BMMs+platelets mixture (designated BMM+PLT^DiD^) and BMMs conjugated with platelets via a click reaction (designated BMM‐PLT^DiD^) were intravenously injected into SCI mice. Both BMM^DiD^ and BMM‐PLT^DiD^ resulted in greater fluorescence signals in the spinal cord than BMM+PLT^DiD^ did 6 and 24 h after injection, which suggests that BMMs had a superior ability to accumulate at the injury site. Moreover, the simple mixture of BMM and PLT^DiD^ did not increase the accumulation of PLT^DiD^ in the spinal cord (Figure 6F,G). This clearly indicates that the transport of the conjugated platelets to the injury site is primarily mediated by the targeting capability of macrophages, a feat that platelets alone cannot accomplish. We also found that, compared with those in the BMM+PLTs group, the number of transplanted platelet mitochondria in macrophages was greater in the BMM‐PLTs group, which suggests that the combined delivery system effectively improved mitochondrial transplantation in vivo (Figure 6H,I), and the BMM‐PLTs group presented increased macrophage ATP content (Figure 6J). Next, Cy5‐labeled DNA was used to further verify targeting at the cellular level. DNA^Cy5^ was effectively internalized by macrophages, as evidenced by the red puncta (Cy5) inside the cells, indicating that the BMM‐PLT complex successfully delivered NPs into the injury site (Figure 6K). Furthermore, the expression of PPARγ was distinctly increased in macrophages treated with M‐P‐NPs@PPARγ but not in macrophages treated with M‐P‐NPs@NC (Figure 6L). Therefore, the results of radiography and histological examination suggest that M‐P‐NPs@PPARγ were able to efficiently deliver DNA and mitochondria into macrophages in vivo.

### 2.6 M‐P‐NPs@PPARγ Facilitates Locomotor Functional Recovery

**Figure 7:**
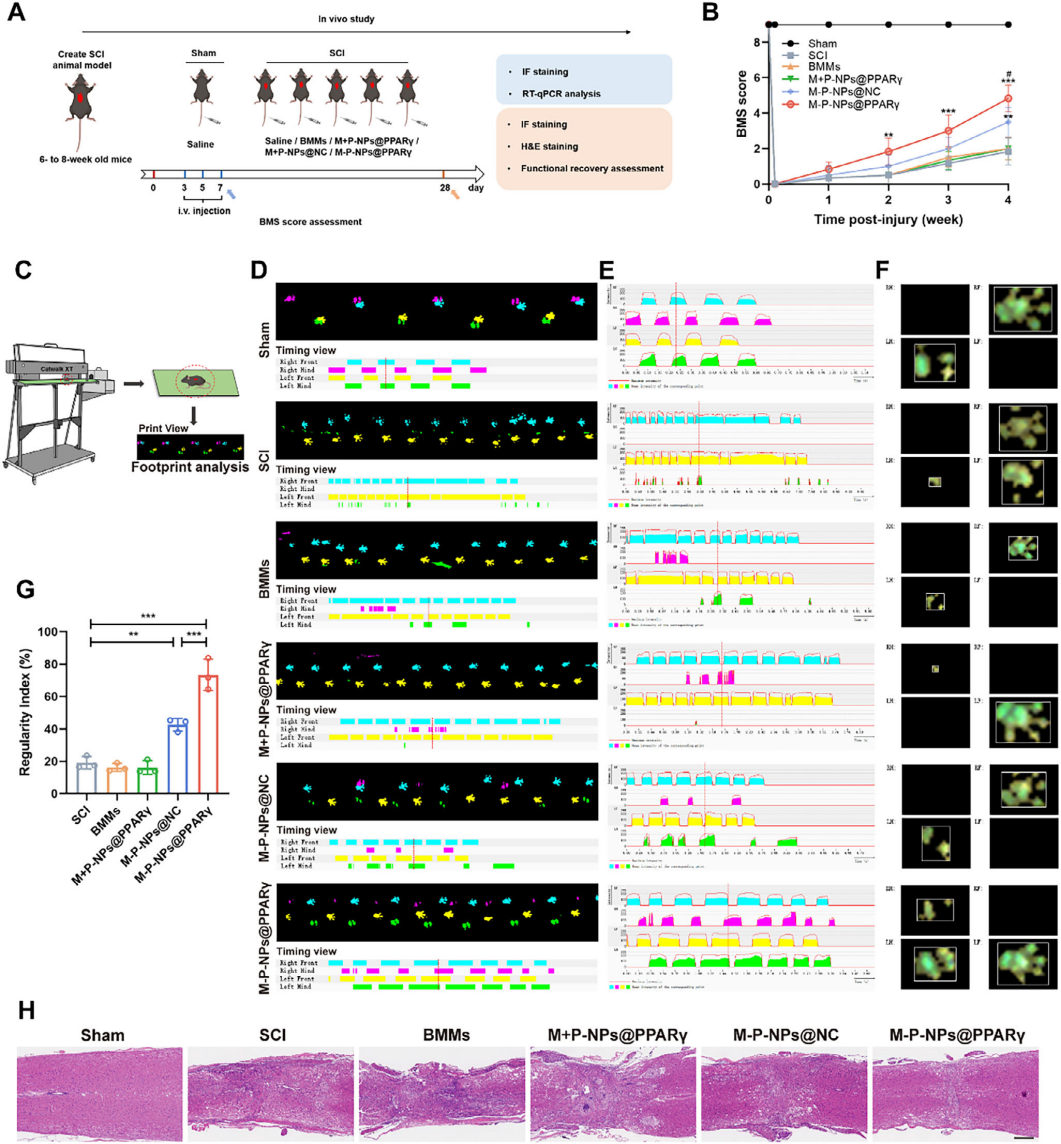
M‐P‐NPs@PPARγ facilitate hindlimb locomotor recovery. A) Schematic illustration of the process and group division of the animal experiments. B) Hindlimb locomotor recovery in mice was evaluated using BMS scoring. Compared with those in the SCI group, the locomotor scores of the M‐P‐NPs@NC and M‐P‐NP@PPARγ group were improved after injury (n = 6). Compared with the SCI group, the M‐P‐NP@NC group also had improved BMS locomotor scores. C) Schematic diagram showing data collection from the CatWalk gait test. D‐F) Representative CatWalk gait of print view, timing view, area chart of print intensity, and foot view for each group. We marked one single specific time point in the images (E) of the area chart of print intensity and provided their associated foot views in images (F). G) Regularity index (%), a measure of interlimb coordination based on the CatWalk gait data (n = 3). H) Representative H&E images 28 days after SCI. Scale bar, 400 µm. All data are depicted as means ± SD; ^#^ *p* < 0.05, **p < 0.01, or ***p < 0.001.

To further investigate the protective and therapeutic effects in vivo, SCI model mice were intravenously injected with saline, BMMs, M+P‐NPs@PPARγ, M‐P‐NPs@NC, or M‐P‐NPs@PPARγ to evaluate the therapeutic effects (**Figure** **7**A). The Basso Mouse Scale (BMS) score was used to assess mouse functional recovery after SCI. Each mouse exhibited normal locomotor activity before injury (score of 9) and complete paralysis (score of 0) postsurgery (Figure 7B). The results revealed that the M‐P‐NPs@PPARγ groups presented significantly higher BMS scores 4 weeks after SCI. Despite M‐P‐NPs@NC group facilitated locomotor functional recovery after SCI, this improvement was markedly lower than that of the M‐P‐NPs@PPARγ group. To better investigate the gait changes in detail, we utilized the CatWalk XT System to record the footprints of the mice 4 weeks postsurgery (Figure 7C). The recorded parameters included the time it took for each paw to contact the ground (Figure 7D), the area chart of print intensity (Figure 7E), and the foot view (Figure 7F). No obvious front limb abnormalities were observed across the groups. The hindlimbs of the SCI, BMMs and M+P‐NPs@PPARγ group mice could not be lifted off the ground, resulting in an inability to test the hindlimbs. Mice treated with M‐P‐NPs@NC displayed supportive plantar placement but with uncoordinated stepping. However, mice treated with M‐P‐NPs@PPARγ exhibited coordinated crawling using their hind limbs, whereas mice in the SCI, BMMs and M+P‐NPs@PPARγ groups still exhibited dragging and uncoordinated crawling. The ratio of regular steps to total steps (regulatory index) in the M‐P‐NPs@PPAR groups was markedly greater than that in the other groups (Figure 7G). The histopathological results indicated that the lesion area of the M‐P‐NPs@PPARγ group was occupied with abundant regenerated tissues, indicating that M‐P‐NPs@PPARγ better facilitated tissue regeneration after SCI (Figure 7H). Collectively, the results provided evidence that M‐P‐NPs@PPARγ treatment enabled better locomotor functional recovery after SCI.

### 2.7 M‐P‐NPs@PPARγ Promote Axonal Regeneration and Remyelination After SCI

**Figure 8:**
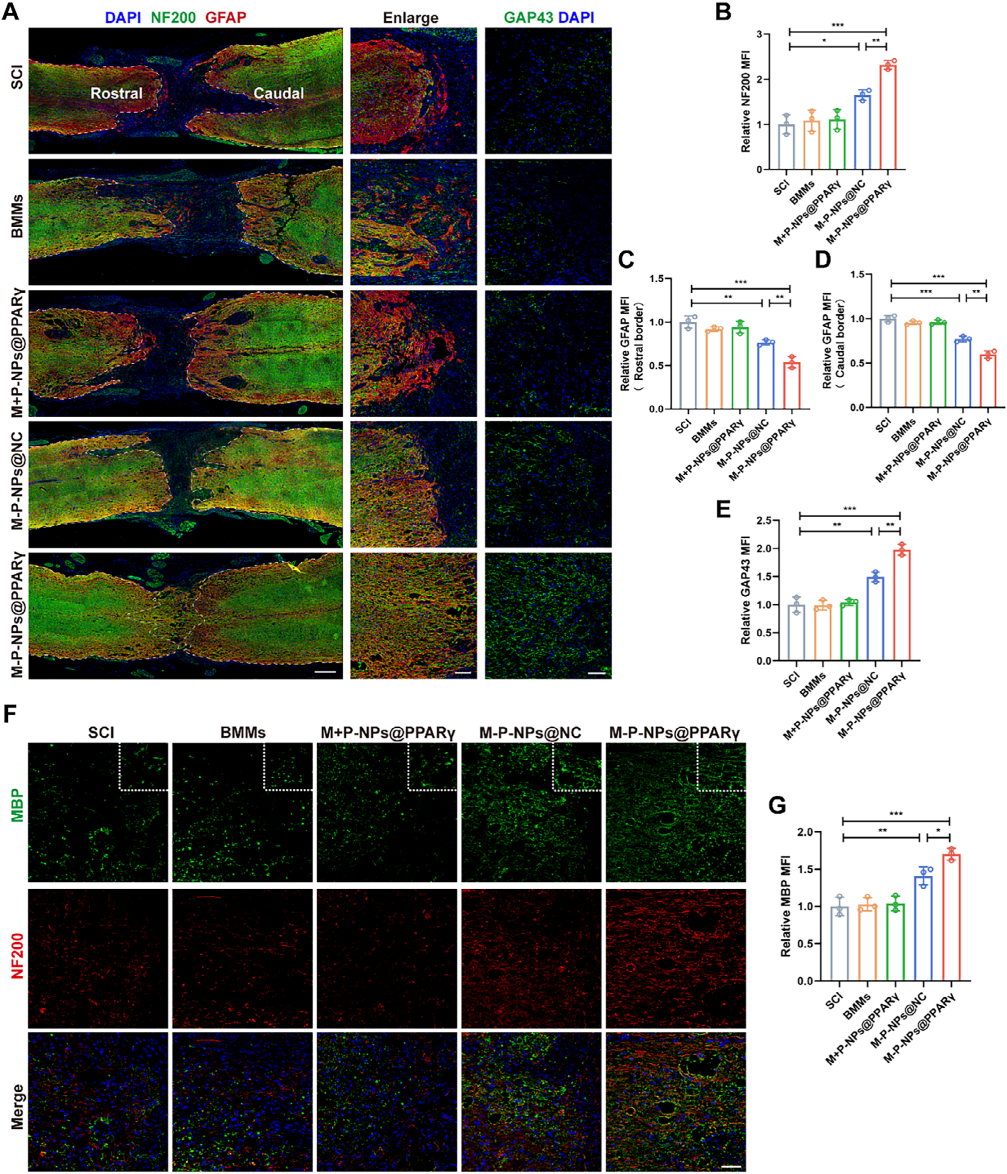
M‐P‐NPs@PPARγ induce neural regeneration and remyelination after SCI. A) Immunofluorescence images showing GFAP (red) and NF200 (green) expression in each group after SCI. Scale bar, 300 µm. Enlarged scale bar, 100 µm. The white dashed lines indicate the lesion border. Immunofluorescence images showing GAP43 (green) and DAPI (blue) after SCI in each group. Scale bar, 50 µm. B) Quantification of the fluorescence intensity of NF200. The fluorescence intensity was measured at the injury site center (n = 3). C,D) Quantification of GFAP fluorescence intensity. The fluorescence intensity was measured at the rostral and caudal borders of the injury site (n = 3). E) Quantification of GAP43 fluorescence intensity (n = 3). F) Myelinated axon regeneration was evaluated in each group. Green fluorescence represents the MBP oligodendrocyte marker. Red immunofluorescence represents the NF200 axonal marker. Scale bar, 50 µm. G) Quantification of MBP fluorescence intensity. The fluorescence intensity was measured at the injury site center (n = 3). All data are depicted as means ± SD; *p < 0.05, **p < 0.01, or ***p < 0.001.

To further confirm the mechanisms of injured spinal cord repair at the cellular level, we used neurofilament (NF200) to label axons and GFAP to label astrocytes. There was no obvious axon sprouting into the middle of the lesion site in the SCI group (**Figure** **8**A). Furthermore, in the SCI, BMMs and M+P‐NP@PPARγ groups, GFAP‐positive astrocytes were located in the rostral and caudal regions and formed a glial scar, which prevented axonal outgrowth from crossing the lesion border. In contrast, axonal regrowth was present in the rostral and caudal regions of the lesion site in a directional arrangement in the M‐P‐NPs@NC and M‐P‐NP@PPARγ groups. Moreover, the middle of the lesion site was filled with NF200‐positive axons in the M‐P‐NPs@PPARγ group (Figure 8A–D). We further performed IF staining for GAP43, which is a widely used neuroprotective protein. The expression of GAP43 had a similar trend to that of NF200 (Figure 8A,E). These results demonstrate that M‐P‐NPs@PPARγ facilitated axon regeneration, providing a cellular basis for functional recovery. In addition, the M‐P‐NPs@NC and M‐P‐NP@PPARγ groups exhibited significantly continuous myelin basic protein (MBP)‐positive signals across the lesion area. Although some myelin sheaths were visible at the injured area in the M‐P‐NP@NC group, they were markedly less visible than those in the M‐P‐NPs@PPARγ group (Figure 8F,G). These results suggest that M‐P‐NPs@PPARγ promoted remyelination of axons, which is also critical for locomotor recovery after SCI.

### 2.8 M‐P‐NPs@PPARγ Mediates Lipid Homeostasis in Macrophages and Enables Optimal Efferocytosis In Vivo

**Figure 9:**
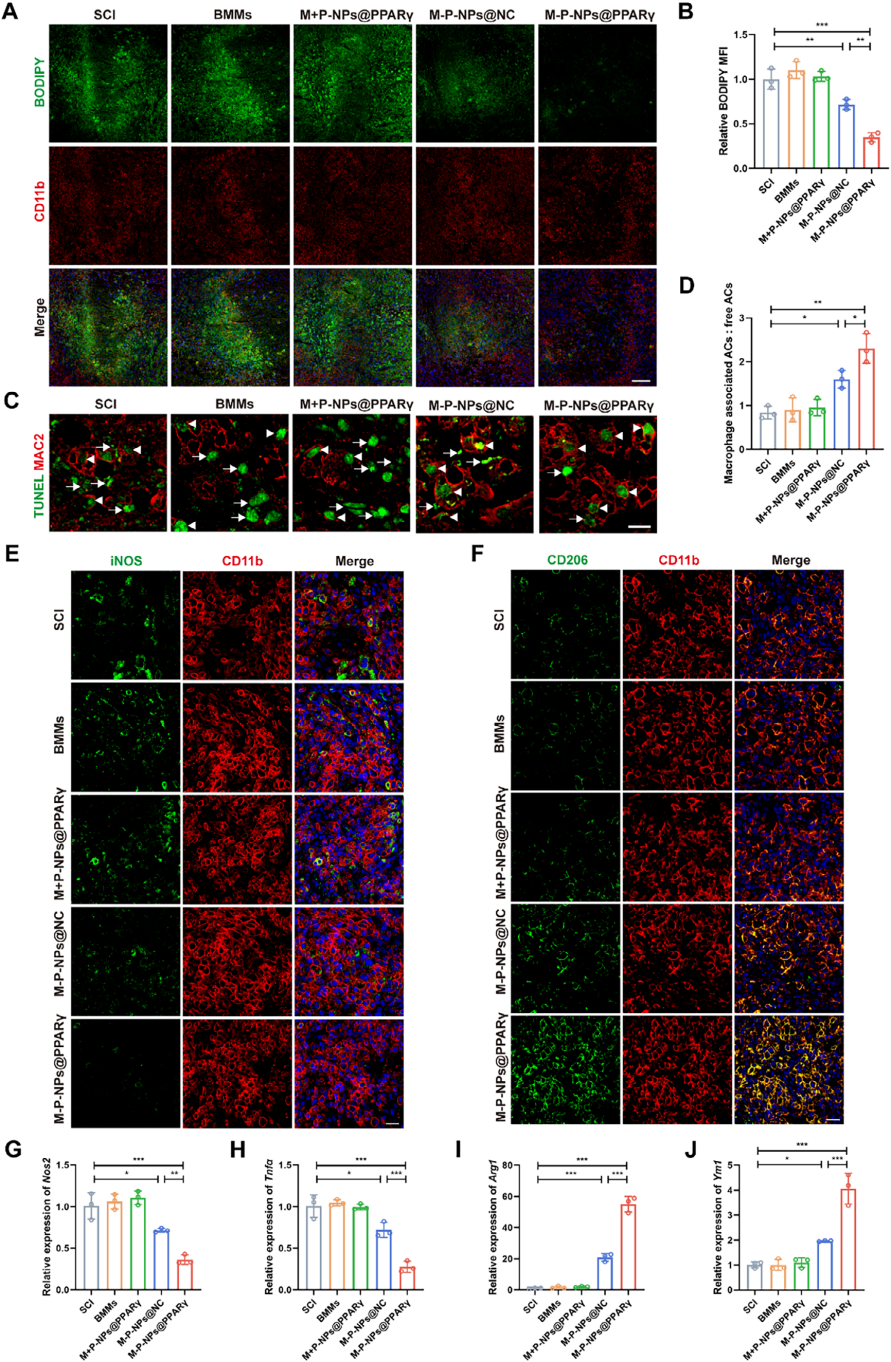
Pro‐efferocytotic M‐P‐NPs@PPARγ promoted macrophage anti‐inflammatory polarization. A) The presence of lipid droplets (BODIPY, green) in macrophages (CD11b, red) at 7 days after SCI in each group. Scale bar, 100 µm. B) Quantification of the fluorescence intensity (BODIPY level) from (A) (n = 3). C) Representative confocal microscopy analysis to evaluate efferocytosis in vivo. MAC2, red; TUNEL, green. Arrowheads and arrows depict macrophage‐associated and free TUNEL^+^ cells, respectively. Scale bar, 10 µm. D) Quantification of efferocytosis (macrophage‐associated ACs: free ACs) in sections from the mice in each group (n = 3). E) Coimmunofluorescence images showing iNOS (green) and CD11b (red) expression in each group after SCI. Scale bar, 25 µm. F) Coimmunofluorescence images showing CD206 (green) and CD11b (red) expression in each group after SCI. Scale bar, 25 µm. G–J) mRNA expression of *Nos2*, *Tnfα*, *Arg1* and *Ym1* after SCI in each group (n = 3). All data are depicted as means ± SD; *p < 0.05, **p < 0.01, or ***p < 0.001.

Given that foam macrophages have impaired phagocytic ability and produce proinflammatory cytokines, early inhibition of harmful foam macrophage formation can provide a beneficial microenvironment for injured spinal cord repair. M‐P‐NPs@PPARγ treatment resulted in fewer lipid droplets in the macrophages, which suggested that M‐P‐NPs@PPARγ‐regulated lipid homeostasis plays a role in foam cell formation inhibition (**Figure** **9**A,B). To further investigate the role of M‐P‐NPs@PPARγ treatment in regulating the engulfment of dead cells, we compared the phagocytic ability of macrophages after SCI. As shown in Figure 9C, the M‐P‐NPs@PPARγ presented a greater ratio of macrophage‐associated ACs to free ACs, indicating improved efferocytosis (Figure 9D). Furthermore, compared with the other treatments, M‐P‐NPs@PPARγ treatment decreased the number of M1 macrophages, presented as iNOS^+^ and CD11b^+^ cells, and increased the number of CD206^+^ and CD11b^+^ M2 macrophages (Figure 9E,F), further supporting the effect of M‐P‐NPs@PPARγ in inducing M2 macrophage polarization. The mRNA expression of *Nos2* and *Tnfα* was significantly reduced, and the mRNA expression of *Arg1* and *Ym1* was upregulated in the M‐P‐NPs@PPARγ treatment group (Figure 9G–J). Moreover, the in vivo biosafety of M‐P‐NPs@PPARγ was also verified (Figure S6A, Supporting Information). On the basis of these results, we verified that platelet treatment and maintenance of lipid homeostasis synergistically contributed to improving efferocytosis and altering macrophages toward an anti‐inflammatory phenotype.

## 3 Conclusion

In summary, we devised an innovative strategy that synergistically integrates mitochondrial transfer with gene delivery to restore macrophage efferocytosis following SCI. We proved that anucleate platelet‐derived mitochondria possess superior resilience to oxidative damage and can restore the energy status of macrophages by transferring mitochondria under cellular stress conditions. This treatment strategy enables the maintenance of mitochondrial activity and effectively induces the expression of genes that regulate lipid homeostasis within the injury microenvironment. Therefore, this cell‐assembly‐mediated mitochondria and gene delivery system is generalizable. The targeting capacity of BMMs, the functional mitochondrial transfer from platelets, and the ROS‐responsive release of NPs that promote gene expression collectively provide a range of functionalities and advantages for the treatment of diverse tissue injuries.

## 4 Experimental Section

### Bioinformatic Analysis of the Transcriptomic Dataset

Transcriptome data of platelet mitochondria were obtained from the GEO database under the accession number GSE206543. Briefly, following preprocessing, differential expression analysis was conducted using DESeq2, and this work identified differentially expressed genes associated with platelets and performed enrichment analysis using the DAVID online tool. Furthermore, this work conducted gene set enrichment analysis (GSEA) on the dataset to detect the enrichment of potential pathways.^[^ ^20^ ^]^

### Proteomic Sample Preparation

BMMs and platelets (1 × 10^7^ cells) were stimulated with either 200 µM H_2_O_2_ or a control condition for 8 h. Proteomic sample preparation was performed using a Mitochondria Isolation Kit (Miltenyi Biotec, Germany). Briefly, 1–2 mL of lysate was used to resuspend the sample. Ice‐cold 1× separation buffer was added to a final volume of 10 mL. Next, 50 µL of anti‐TOM22 microbeads was added to magnetically label the mitochondria, which were subsequently incubated for 1 h in a refrigerator (2–8 °C) with gentle shaking. After incubation, the magnetically labeled mitochondria were isolated using a MACS separator. For sample preparation, DTT (with a final concentration of 40 mM) was added to each sample and mixed at 600 rpm for 1.5 h (37 °C). After the samples cooled to room temperature, IAA was added to the mixture at a final concentration of 20 mM to block reduced cysteine residues, and the samples were incubated for 30 min in the dark. Next, the samples were transferred to the filters (Microcon units, 10 kDa). The filters were washed with 100 µL of UA buffer three times and then with 100 µL of 25 mM NH_4_HCO_3_ buffer twice. Finally, trypsin was added to the samples (the trypsin:protein (wt/wt) ratio was 1:50) and incubated at 37 °C for 15–18 h (overnight), and the resulting peptides were collected as a filtrate. The peptides of each sample were desalted on C18 cartridges (Empore SPE Cartridges MCX, 30 µm, Waters), concentrated by vacuum centrifugation and reconstituted in 40 µL of 0.1% (v/v) formic acid. The peptide content was estimated from the UV light spectral density at 280 nm.

### Mass Spectrometry Data Analysis

The peptides from each sample were analyzed via an OrbitrapTM AstralTM mass spectrometer (Thermo Scientific) connected to a Vanquish Neo system liquid chromatograph (Thermo Scientific) in data‐independent acquisition (DIA) mode. Precursor ions were scanned at a mass range of 380–980 m/z, the MS1 resolution was 240 000 at 200 m/z, the normalized AGC target was 500%, and the maximum IT was 5 ms. A total of 299 windows were set for DIA mode in MS2 scanning, the isolation window was 2 m/z, the HCD collision energy was 25 ev, the normalized AGC target was 500%, and the maximum IT was 3 ms. The DIA data was analyzed with DIA‐NN 1.8.1. The main software parameters were set as follows: the enzyme was trypsin, the maximum number of missed cleavages was 1, the fixed modification was carbamidomethyl (C), and the dynamic modifications were oxidation (M) and acetylation (protein N‐term). All reported data were based on 99% confidence for protein identification as determined by a false discovery rate (FDR) ≤ 1%.

### Cell Culture

Fresh BMMs from 6‐ to 8‐week‐old C57BL/6J mice were isolated as previously reported.^[^ ^35^ ^]^ Briefly, the femur‐ and tibia‐derived bone marrow was flushed and then cultured in α‐minimal essential medium (α‐MEM) supplemented with 10% fetal bovine serum (FBS; Gibco, U. S. A) and 1% penicillin G‐streptomycin (complete α‐MEM). To obtain pure BMMs, nonadherent cells were collected and cultured in complete α‐MEM plus M‐CSF (25 ng mL^−1^, R&D Systems, U. S.A.). After 3 days of culture, the attached cells were used for experiments. Jurkat and RAW 264.7 cells were purchased from the Cell Bank of the Type Culture Collection of the Chinese Academy of Sciences, Shanghai Institute of Cell Biology, Chinese Academy of Sciences. Jurkat and RAW 264.7 cells were cultured in RPMI 1640 (Gibco, U. S. A) supplemented with 10% FBS and 1% penicillin G‐streptomycin. All the cells were incubated in a humidified atmosphere containing 95% air and 5% CO_2_ at 37 °C.

### FACS

Single‐cell suspensions were prepared from the mouse spinal cord using the Percoll gradient isolation method. Briefly, the mice were anesthetized and intracardially perfused with sterile 0.9% NaCl. The spinal cord was removed, minced into small pieces, and then treated with 10 mg mL^−1^ papain (Sigma‒Aldrich, Australia) and 10 µg mL^−1^ DNase I (Sigma‒Aldrich, Australia) for 20 min at 37 °C. The cell suspensions were passed through a 40‐µm cell strainer and fractionated on a 30%/70% discontinuous Percoll gradient at 500 × g for 30 min. Mononuclear cells were collected from the interface. All the isolated cells were stained with the following antibodies and the appropriate isotype controls: anti‐CD11c (562 782 BD Biosciences), anti‐Ly6G (11‐9668‐82 eBioscience), anti‐CD11b (17‐0112‐83 eBioscience) and anti‐CD45 (45‐0451‐82 eBioscience). FACS was performed using a FACSAria III sorter and FACSDiva software (BD Biosciences). The macrophages were gated as Ly6G^−^CD11c^−^CD11b^+^CD45^high^ and sorted for RT‒qPCR and determination of ATP levels via an ATP assay kit (S0026, Beyotime, China) according to the manufacturer's protocol.

### Platelet Preparation and Evaluation of Mitochondrial Transfer from Platelets to Macrophages

Platelet‐rich plasma (PRP) was prepared using a previously reported method.^[^ ^36^ ^]^ In brief, whole blood samples were collected from healthy volunteers and mixed with acid citrate dextrose solution A (ACD‐A) anticoagulant (1 mL ACD‐A/9 mL blood). After centrifugation at 100 × g for 15 min, the platelet‐containing plasma was collected and centrifuged at 800 × g for 20 min. The supernatant plasma was discarded, and the platelet pellet was resuspended at an average concentration of 10^9^ platelets mL^−1^. To inhibit mitochondrial respiration, the platelets were incubated for 3 h with 2 µM rotenone (R8875, Sigma‒Aldrich) and 2 µM antimycin A (MS0070, Shanghai Maokang Biotechnology Co., Ltd.), which are inhibitors of complex I and complex III of the respiratory chain, respectively. Prior to use, untreated and ROT/AA‐treated platelets were rinsed three times in phosphate‐buffered saline (PBS, pH = 7.4) containing 1 µM PGE1 to avoid platelet aggregation. To evaluate the transfer of mitochondria from platelets to BMMs, BMMs were treated with FITC‐labeled wheat germ agglutinin (FITC‐WGA, 5 µg mL^−1^, MP6325, Shanghai Maokang Biotechnology Co., Ltd.) and 200 µM H_2_O_2_ prior to their exposure for 12 h to platelets previously labeled with MitoTracker Deep Red FM (40 nM, 40743ES50, Yeasen, Shanghai). The presence of platelet‐derived mitochondria in BMMs was detected by confocal fluorescence microscopy (Nikon, A1 PLUS, Tokyo, Japan). ATP levels were measured with an ATP assay kit.

To further prove the transfer of mitochondria from platelets to macrophages in vivo, platelets labeled with MitoTracker Deep Red were directly injected into the injured region of the mice 1 day after SCI. Spinal cord samples were obtained 1 day after injection. Macrophage suspensions were prepared from the mouse spinal cord using FACS. The transplanted mitochondria were examined by flow cytometry analysis of macrophages.

### Staining of Infiltrating Cells in Injured Tissue

Femoral defects were created in 10‐week‐old C57BL/6J mice following an established procedure with slight modifications.^[^ ^37^ ^]^ Briefly, after anesthesia was induced, the mice were dissected, and the periosteum was peeled back to allow access to the bone, exposing the shaft of the left femur. A 1 mm mid‐diaphyseal defect was then created using a 1 mm biopsy punch (Miltex). The defect site was irrigated with saline to remove bone debris. The wounds were closed with sutures, and the mice were injected with buprenorphine to provide pain relief after surgery. All the mice were humanely euthanized 1, 3, 5 or 7 days after femoral defects or SCI, and femur and spinal cord tissues were obtained. All the tissues were postfixed in 4% PFA for 1 day. The femurs were decalcified in 14% EDTA (Sigma‒Aldrich, Germany) for 7 days at 37 °C. All the tissues were subsequently embedded in paraffin for sectioning. The samples were then incubated with primary antibodies against CD41 and MAC2 overnight at 4 °C. The sections were washed three times with PBST and incubated with Alexa Fluor 488‐conjugated goat anti‐rabbit secondary antibodies and Alexa Fluor 594‐conjugated goat anti‐mouse secondary antibodies for 1 h at 37 °C. The sections were rinsed three times with PBST and incubated with DAPI Fluoromount‐G for 10 min. All the images were captured via a confocal fluorescence microscope. The fluorescence data were analyzed with ImageJ software.

### Preparation and Characterization of the NPs

**Scheme 1:**
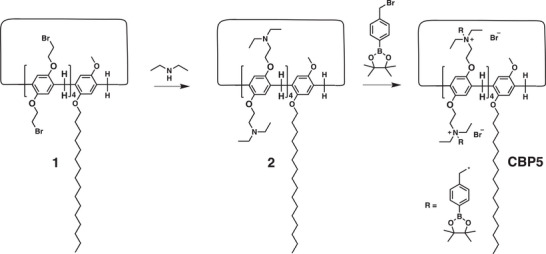
Synthetic route to CBP5.

CBP5 was synthesized as reported (an invention disclosure of patent no. CN117720563A). Briefly, as shown in the schematic diagram, compounds 1 and 2 were synthesized according to previously reported methods.^[^ ^38^, ^39^ ^]^ For CBP5, 2‐(4‐(bromomethyl)phenyl)‐4,4,5,5‐tetramethyl‐1,3,2‐dioxaborolane (0.98 g, 3.30 mmol) was added to a solution of compound 2 (0.48 g, 0.30 mmol) in acetonitrile (50 mL). The mixture was stirred and refluxed for 24 h. Afterward, the mixture was cooled to room temperature and then concentrated by evaporation. Excess ether was poured into the concentrated solution, and the resulting precipitate was collected by filtration. The solid was washed with ice‐cold ether three times to remove the residual reactant. CBP5 (1.20 g, yield 87%) was obtained as a white solid after drying under vacuum (**Scheme** **1**).

Plasmid DNA was dissolved in HEPES buffer solution (10 mM, pH 7.4) at a concentration of 40 µg mL^−1^. PBS was used to dilute CBP5 at various concentrations according to the preset N/P ratios (N/P = 10, 15, 20, 25, and 30). Subsequently, equal volumes of the CBP5 solution were added to the DNA mixture. The mixture was immediately vortexed for 20 s and then statically incubated for 30 min to obtain the NPs. The sizes and zeta potentials of the NPs were measured via dynamic light scattering (DLS) (Malvern, UK). The encapsulation efficiency of NPs with various N/P ratios and ROS responsiveness were assessed via gel retardation assays. NPs with different N/P ratios were treated with or without 5 mM H_2_O_2_ at 37 °C for 1 h and then subjected to electrophoresis on a 1% agarose gel at 100 V for 30 min. Gel Red (Biotium) was used in the agarose gel for DNA detection. The morphology of the NPs stained with water‐soluble phosphotungstic acid was visualized using a transmission electron microscope (JEM‐1400plus).

### DNA Transfection Efficiency

To evaluate luciferase gene transfection, the pGL4.13 (Promega) plasmid vector encoding the luciferase reporter gene was used. The cells were cultured at a density of 1.5 × 10^5^ cells per well in a 96‐well plate. The medium was replaced with fresh medium (containing 0 or 10% FBS). NP solutions (25 µL) were added at a dose of 1 µg of DNA per well, and the cells were incubated for 4 h. The medium was subsequently replaced with 200 µL of fresh complete medium. The cells were cultured for an additional 44 h. The expression level of the luciferase plasmid was measured according to the manufacturer's instructions (Promega). The protein concentration of the cell lysis mixture was evaluated with a BCA assay kit. The luciferase activity was normalized with respect to the protein concentration (relative luciferase luminescence units per milligram of protein).

### Preparation and Characterization of Platelets and P‐NPs

After the platelets were isolated, the cells were suspended in PBS containing 1 µM PGE1. To prepare the P‐NPs, freshly prepared NPs were slowly added to the platelet mixture at a ratio of 1:4 (v/v), and the mixture was inverted three times following incubation in a shaker at 60 rpm at room temperature. Then, the P‐NPs were separated via centrifugation (800 × g, 10 min) and rinsed with PBS containing 1 µM PGE1. P‐NPs were resuspended in PBS containing 1 µM PGE1 before use. The sizes and zeta potentials of the platelets and P‐NPs were measured via DLS.

To further determine the loading of the NPs into the platelets, the DNA was labeled with Cy5 (Mirus Bio) according to the manufacturer's instructions and mixed with CBP5 at a 20 N/P ratio. The platelets were stained with 5 µg mL^−1^ FITC‐WGA for 10 min at 37 °C before being loaded with prepared DNA^Cy5^. After rinsing with PBS containing 1 µM PGE1, P^FITC^‐NPs@DNA^Cy5^ were observed and imaged via confocal fluorescence microscopy.

SEM was used to observe the morphology of the P‐NPs before and after incubation with 0.5 U mL^−1^ thrombin (Th). Briefly, P‐NPs were pretreated with or without 0.5 U mL^−1^ Th for 30 min at 37 °C. were fixed with 3.5% glutaraldehyde for 4 h, washed three times with PBS, and sequentially dehydrated with various concentrations of ethanol (30%, 50%, 70%, 85% and 95% each time for 15 min and 100% twice for 30 min). The samples were treated with tert‐butanol, allowed to dry under vacuum, and observed via SEM (TM‐1000, Hitachi, Tokyo, Japan) after gold spraying. The expression of specific markers on NPs and P‐NPs with or without Th treatment and NPs were determined by WB.

To investigate the stability of the P‐NPs, freshly prepared P‐NPs@DNA^Cy5^ were suspended in PBS and serum‐containing medium with or without 0.5 U mL^−1^ Th and incubated in a shaker at 90 rpm at 37 °C. The mixtures were subsequently centrifuged (800 × g, 5 min) at predetermined time intervals. The supernatant was collected to measure the DNA^Cy5^ concentration via spectrofluorimetry (λ_ex_ = 633 nm, λ_em_ = 678 nm).

### WB Analysis

Proteins were extracted from the platelets and BMMs using RIPA buffer containing 1 mM PMSF (Boster, China). The cell lysates were subsequently centrifuged at 12 000 rpm for 10 min at 4 °C, after which the supernatants were collected. The protein concentrations were quantified with a BCA protein assay kit (Beyotime, China). Proteins were separated by SDS‒PAGE and transferred to a polyvinylidene fluoride (PVDF) membrane (Millipore, Shanghai, China). After being blocked with 5% nonfat milk for 2 h, the membrane was washed with Tris‐buffered saline with 0.1% Tween‐20 (TBST) three times for 5 min each. The membranes were subsequently incubated with primary antibodies at 4 °C overnight. The membranes were incubated with secondary antibodies for 1 h at room temperature after being washed with TBST three times for 5 min each. Finally, the signals were visualized with a ChemiDoc Touch imaging system (Bio‐Rad, USA). The antibodies used are listed in Table S2, Supporting Information. All of the above experiments were performed in triplicate.

### Cellular Uptake Assay

The cellular uptake of the NPs and P‐NPs with or without Th was examined by confocal microscopy and flow cytometry of the BMMs. DNA was labeled with Cy5, and NPs@DNA^Cy5^ and P‐NPs@DNA^Cy5^ were prepared as described above. BMMs were seeded onto glass‐bottom Petri dishes at 1 × 10^5^ cells per dish in 2 mL of cell culture medium before use. Subsequently, solutions (50 µL) of NPs or P‐NPs with or without Th were added at a dose of 1 µg of DNA per dish. The nuclei were stained with two drops of Hoechst 33 342 (Molecular Probes, Carlsbad, CA) per milliliter of medium for 15 min after 4 h of incubation. The cells were subsequently analyzed using flow cytometry to determine the percentage of Cy5‐positive cells (1 × 10^4^ cells were counted per treatment). Images were obtained via a confocal fluorescence microscope.

### Subcellular Distribution Analysis

DNA^Cy5^ was complexed with CBP5 at an N/P ratio of 20 at room temperature before use, and P‐NPs were prepared as described above. Solutions of P‐NPs labeled with fluorescent dyes were treated at a dose of 1 µg of DNA per dish. After a timed incubation, the medium was replaced with fresh medium. To label the lysosomes, the cells were further incubated with 200 nM LysoTracker Green (Molecular Probes, Carlsbad, CA) for 15 min, and then the nuclei were stained with two drops of Hoechst 33 342 for 15 min. The cells were observed via confocal fluorescence microscopy.

### Real‐Time Quantitative Polymerase Chain Reaction

Total RNA was extracted from lysed BMMs using TRIzol reagent (Invitrogen, Shanghai, China) and an RNA purification kit (CW0581, Kangwei) according to the manufacturer's instructions. The cDNA was reverse transcribed using the MMLV reverse transcription reagent (Takara). GAPDH was used as an internal reference. BMMs subjected to the respective treatments were stimulated with lipopolysaccharide and interleukin‐4 and then assayed mRNA by RT‐qPCR. Real‐time PCR was performed using a SYBR Master Mix qPCR kit (Takara). The primers used are listed in Table S1, Supporting Information. All the experiments were performed in triplicate.

### In Vitro PPARγ Overexpression

BMMs cultured in six‐well plates (2 × 10^5^ cells per well) were stimulated with H_2_O_2_ for 8 h. Different treatments were added to the cells for 4 h. Then, the medium was replaced with the corresponding complete medium, and the cells were incubated for another 44 h. PPARγ expression was measured via RT**‒**qPCR and WB analysis.

### In Vitro Phagocytosis Assay

To determine the efferocytosis of BMMs, Jurkat cells were labeled with 2′,7′‐bis‐(2‐carboxyethyl)‐5‐(and‐6)‐carboxyfluorescein (BCECF‐AM) and irradiated under a 254‐nm UV lamp for 15 min, followed by incubation under normal cell culture conditions for 2 h to obtain Acs.^[^ ^11^ ^]^ For macrophages subjected to relevant treatments, BCECF‐AM–stained ACs were added at a ratio of 1:5 to 10 and incubated for 2 h in DMEM containing 2% FBS. The cells were then rinsed with PBS and fixed with 4% PFA. Then, the macrophages were stained with rhodamine‐coupled phalloidin for 1 h at room temperature. The cells were washed three times with PBS and treated with DAPI Fluoromount‐G for 10 min. Images were captured via a confocal fluorescence microscope.

For the myelin debris uptake assay, myelin debris was obtained as previously described.^[^ ^40^ ^]^ The endotoxin concentration of myelin debris was below the detection limit of the Limulus Amebocyte Lysate assay. Myelin debris was used at a concentration of 1 mg mL^−1^ for cell culture throughout this study. BMMs were seeded onto glass‐bottom Petri dishes at 1 × 10^5^ cells per dish in 2 mL of cell culture medium before use. Myelin debris was added to the BMM cultures for the indicated time periods. To measure lipid droplets, the cells were washed with PBS and fixed with 4% PFA. After immunostaining with the F4/80 antibody, the cells were incubated with 1 mg mL^−1^ BODIPY (D3922, Invitrogen) and Alexa Fluor 594‐conjugated goat anti‐mouse secondary antibodies for 1 h at 37 °C, followed by three washes. Myelin debris uptake was analyzed by confocal fluorescence imaging. The fluorescence data were analyzed with ImageJ software.

### RNA‐Seq and Bioinformatic Analysis

Total RNA from the BMM (control), H_2_O_2_ and H_2_O_2_+P‐NPs@PPARγ groups (1 × 10^6^ per well) was extracted using TRIzol reagent according to the manufacturer's instructions. The purity of the sample was determined via a NanoPhotometer (IMPLEN, CA, USA), and the concentration and integrity of the RNA samples were detected via an Agilent 2100 RNA nano 6000 assay kit (Agilent Technologies, CA, USA). A total of 1–3 µg of RNA per sample was used as input material for the RNA sample preparations. Sequencing libraries were generated using the VAHTS Universal V6 RNA‐Seq Library Prep Kit for Illumina (NR604‐01/02) following the manufacturer's recommendations, and index codes were added to attribute sequences to each sample. The RNA concentration of the library was measured using a Qubit RNA Assay Kit with a Qubit 3.0 instrument for preliminary quantification, after which the RNA was diluted to 1 ng µL^−1^. Insert size was assessed using the Agilent Bioanalyzer 2100 system (Agilent Technologies, CA, USA). After the insert size reached the expected size, a Bio‐Rad CFX96 fluorescence quantitative PCR instrument was used to accurately quantify the library effective concentration (Library effective concentration > 10 nm). Cluster generation and sequencing were performed on a NovaSeq 6000 S4 platform, using a NovaSeq 6000 S4 Reagent kit V1.5.

### Preparation and Characterization of M‐P‐NPs

BMMs were cultured in 40 µM Ac4GalNAz (Thermo Fisher Scientific)‐containing medium for 72 h. To functionalize the platelets with triple bonds, 1 × 10^6^ platelets were added to 20 µM DBCO‐PEG 4‐NHS ester for 30 min at room temperature for the click reaction, after which the NPs were encapsulated as described above. For the conjugation of platelets or P‐NPs to BMMs, 1 × 10^7^ DBCO‐PEG 4‐NHS ester‐treated platelets stained with rhodamine B were added to 1 × 10^7^ Ac4GalNAz‐treated BMMs and incubated for 45 min at 37 °C. Subsequently, excess 50 µM azide‐PEG was used to quench the additional DBCO on the surface of the platelets for 15 min. After centrifugation at 300 × g for 5 min, the resulting macrophage‒platelet assembly was observed via confocal fluorescence microscopy and SEM. To test the bioactivity of platelets after conjugation with BMMs, M‐P‐NPs were treated with 0.5 U mL^−1^ Th for 30 min at 37 °C and then centrifuged at 400 × g for 5 min. Then, the cells were analyzed using flow cytometry.

### In Vivo Targeting Experiment

The specific injury site targeted by macrophage‒platelet assembly was confirmed using an IVIS Spectrum In Vivo Imaging System. BMMs and platelets were labeled with 1,1′‐dioctadecyl‐3,3,3′,3′‐tetramethylindodicarbocyanine perchlorate (DiD) for 15 min, and BMM^DiD^ and PLT^DiD^ were isolated by centrifugation at 400 × g for 5 min and 800 × g for 10 min, respectively. After washing twice with PBS, the BMM‐PLT^DiD^ assembly was prepared as described above. BMM^DiD^ (BMMs: 1 × 10^6^), a mixture of BMMs and PLT^DiD^ (BMM+PLT^DiD^, BMM/PLT: 1 × 10^6^), and BMM‐PLT^DiD^ (BMM/PLT: 1 × 10^6^; in 200 µL of PBS for each mouse) were injected into the tail veins of the mice 3 days after SCI. Biodistribution analysis of DiD was performed with a 644‐nm excitation wavelength and a 663‐nm filter at 6 and 24 h after injection. Furthermore, the heart, liver, spleen, lung, kidneys and spinal cord were harvested and analyzed in vitro for the distribution of BMM^DiD^, BMM+PLT^DiD^ and BMM‐PLT^DiD^. Living Image software was used to quantify the fluorescence intensity. The method of evaluating the transfer of mitochondria from platelets to macrophages in vivo was as described above.

To further prove specific targeting, M+P‐NPs@DNA^Cy5^ and M‐P‐NPs@DNA^Cy5^ were injected into the tail vein 3 days after SCI. Spinal cord tissue sections were prepared to assess the distributions of M+P‐NPs@DNA^Cy5^ and M‐P‐NPs@DNA^Cy5^ in vivo. Briefly, the spinal cord was obtained 1 day after injection. All the samples were postfixed in 4% PFA for 1 day and embedded in paraffin for sectioning. The samples were then incubated with anti‐CD11b primary antibodies overnight at 4 °C. After primary antibody incubation, the sections were rinsed with PBST for 3 × 7 min and subsequently incubated with Alexa Fluor 594‐conjugated goat anti‐rabbit secondary antibodies for 1 h at room temperature. The sections were washed three times with PBST and incubated with DAPI Fluoromount‐G for 10 min. The images were captured via confocal fluorescence microscopy.

To determine the overexpression of PPARγ in vivo, the spinal cord was collected after 5 days of two injections of M‐P‐NPs@NC or M‐P‐NPs@PPARγ. Then, the macrophages were collected by FACS as described above. The cells were sorted for ATP level detection via RT‒qPCR.

### Spinal Cord Injury In Mice

The animal experiments were approved by the Ethics Committee of the Laboratory Animal Center of Zhejiang University (ZJU20230039) and were performed according to the Guide for the Care and Use of Laboratory Animals. As shown in Figure 7A and 6‐ to 8‐week‐old C57BL/6J mice were randomly divided into 6 groups: (i) the sham group, (ii) the SCI group, (iii) the SCI plus BMMs group, (iv) the SCI plus M+P‐NPs@PPARγ group, (v) the SCI plus M‐P‐NPs@NC group and (vi) the SCI plus M‐P‐NPs@PPARγ group (n = 6 per group). The mice in groups (ii) to (vi) underwent laminectomy on the T10 vertebrae under chloral hydrate anesthesia to expose the thoracic spinal cord. Then, contusion injury was induced using an impactor (RWD, China) with a 1 mm diameter rod dropped at 1.5 m s^−1^, and the depth of injury was 0.5 mm from the surface of the spinal cord, whereas the sham group underwent laminectomy without contusion. 3 days after surgery, the mice in the (iii) to (vi) groups were given BMMs, M+P‐NPs@PPARγ, M‐P‐NPs@NC, or M‐P‐NPs@PPARγ (BMM/PLT: 1 × 10^6^; in 200 µL of PBS for each mouse) via tail vein injection. These mice were subjected to the same treatment on the basis of their groups every 2 days for 1 week, whereas the mice in the sham and SCI groups received tail injections of saline as a control. 4 weeks after surgery, all the mice were humanely euthanized to evaluate the reparative effect of M‐P‐NPs@PPARγ.

### Histological Analysis and Immunofluorescence Staining

Spinal cord tissue samples were obtained 7 and 28 days after injury. To confirm the biosafety of these treatments, the major organs were collected at 28 days after injury. All the samples were postfixed in 4% PFA, washed, and embedded in paraffin to obtain sections. The obtained sections were subsequently subjected to hematoxylin and eosin (H&E) staining. For immunofluorescence staining, the sections were blocked with 5% BSA (Fdbio Science) and then incubated with primary antibodies overnight at 4 °C, followed by incubation with the corresponding secondary antibodies conjugated with Alexa Fluor 488 or Alexa Fluor 594 fluorescent dye and 1 mg mL^−1^ BODIPY for 1 h at room temperature. To evaluate efferocytosis, the sections were stained with TUNEL reagents (Roche) overnight at 4 °C. The sections were washed three times with PBST and incubated with DAPI Fluoromount‐G for 10 min. Images were captured via confocal fluorescence microscopy. The fluorescence data were analyzed with ImageJ software. The antibodies used are listed in Table S2, Supporting Information.

### Functional Recovery Analysis in SCI

Locomotion recovery in the mice was assessed by BMS scores and footprint analysis 4 weeks after SCI. To ensure unbiased observation of behavioral recovery, the behavioral recovery of the mice was evaluated by two independent experimenters who were blinded to the experimental conditions. BMS scores were used to evaluate the behavior of the hindlimbs of the mice before surgery and weekly after SCI. The mice were allowed to walk freely on the grid, and the scores were determined on the basis of hind limb movement function, which ranged from 0 (complete paralysis) to 9 (normal locomotion). The gait was recorded and analyzed with a Catwalk XT 10.6 System (Noldus, Wageningen, Netherlands).^[^ ^41^ ^]^ Briefly, animals traverse a horizontal glass plate as fluorescent light is projected across it. Footprints displace the fluorescent light and are recorded by a camera positioned beneath the walkway. The raw data were collected and analyzed using Catwalk 10.6 software. The software automatically labeled the paw prints. This work selected the regulatory index (regular steps/total steps) parameters for analysis.

### Statistical Analysis

All the experiments were performed with at least 3 replicates. All the quantitative data are expressed as the means ± SD. Furthermore, comparisons of these experimental results were assessed using Student's *t* test or Tukey's multiple comparisons test after one‐way analysis of variance (ANOVA). A result was considered statistically significant when the P values satisfied one of the following conditions: *p < 0.05, **p < 0.01, or ***p < 0.001.

## Conflict of Interest

The authors declare no conflict of interest.

## Supporting information



Supporting Information

## Data Availability

The data that support the findings of this study are available from the corresponding author upon reasonable request.
