# Supplementary material for: A Conjugation Delivery System of Macrophages and Platelet Pharmacytes Promotes Regeneration After Spinal Cord Injury
Source: Adv Sci (Weinh). 2025 Dec 25;13(10):e13474. doi: 10.1002/advs.202513474 (PMC12915203; doi:10.1002/advs.202513474)
Supplement: Supplementary file 1 — Supporting Information [file ADVS-13-e13474-s001.docx]

Supporting Information

**A conjugation delivery system of macrophages and platelet pharmacytes promotes regeneration after spinal cord injury**

*Haoli Wang, Hao Hu, Yijun Li, Lintao Hu, Chenhui Gu, Yiwei Zhu, Jing Huang, Na Li, Shuqi Jiang, Shouyan Zu, Jiachen Xu, Yining Wang, Ke Yang, Pengfei Chen, Liqing Shangguan, Yongcheng Wang, Shunwu Fan*^*^*, Xianfeng Lin^*^, Qingqing Wang^*^*

**Table S1 RT-qPCR primers**

| **Name** | **Forward (5’ to 3’)** | **Reverse (5’ to 3’)** |
| --- | --- | --- |
| Pparg | CTCCAAGAATACCAAAGTGCGA | GCCTGATGCTTTATCCCCACA |
| Mertk | CAGGGCCTTTACCAGGGAGA | TGTGTGCTGGATGTGATCTTC |
| Il10 | ATGGGAGGGGTTCTTCCTTG | GGGGGATGACAGTAGGGGA |
| Nos2 | GCAGCACTTGGATCAGGAAC | ACCATCTCCTGCATTTCTTCC |
| Tnfα | ACCGTCAGCCGATTTGCTAT | CTCCAAAGTAGACCTGCCCG |
| Arg1 | CAGCAAAGCAGACAGAACTAAG | AGAAAGGAACTGCTGGGATAC |
| Ym1 | CTACTCCTCAGAACCGTCAG | GCATTTCCTTCACCAGAACAC |
| Abca1 | GCTTGTTGGCCTCAGTTAAGG | GTAGCTCAGGCGTACAGAGAT |
| Abcg1 | CTTTCCTACTCTGTACCCGAGG | CGGGGCATTCCATTGATAAGG |
| Gapdh | AGGACACTGAGCAAGAGAGG | GTAGCTGGGCCTCTCTCATT |

**Table S2 Antibodies used for Western blots or immunofluorescence staining**

| Antibody | Resource |
| --- | --- |
| CD11b | Abcam |
| MAC2 | Proteintech |
| CD42b | Proteintech |
| CD62p | Proteintech |
| CD41  PPARγ | Proteintech  ABclonal |
| F4/80 | Abcam |
| iNOS | Abcam |
| CD206 | Cell Signaling Technology |
| NF200 | Abcam |
| GFAP | Abcam |
| MBP | Cell Signaling Technology |

**Figure S1**


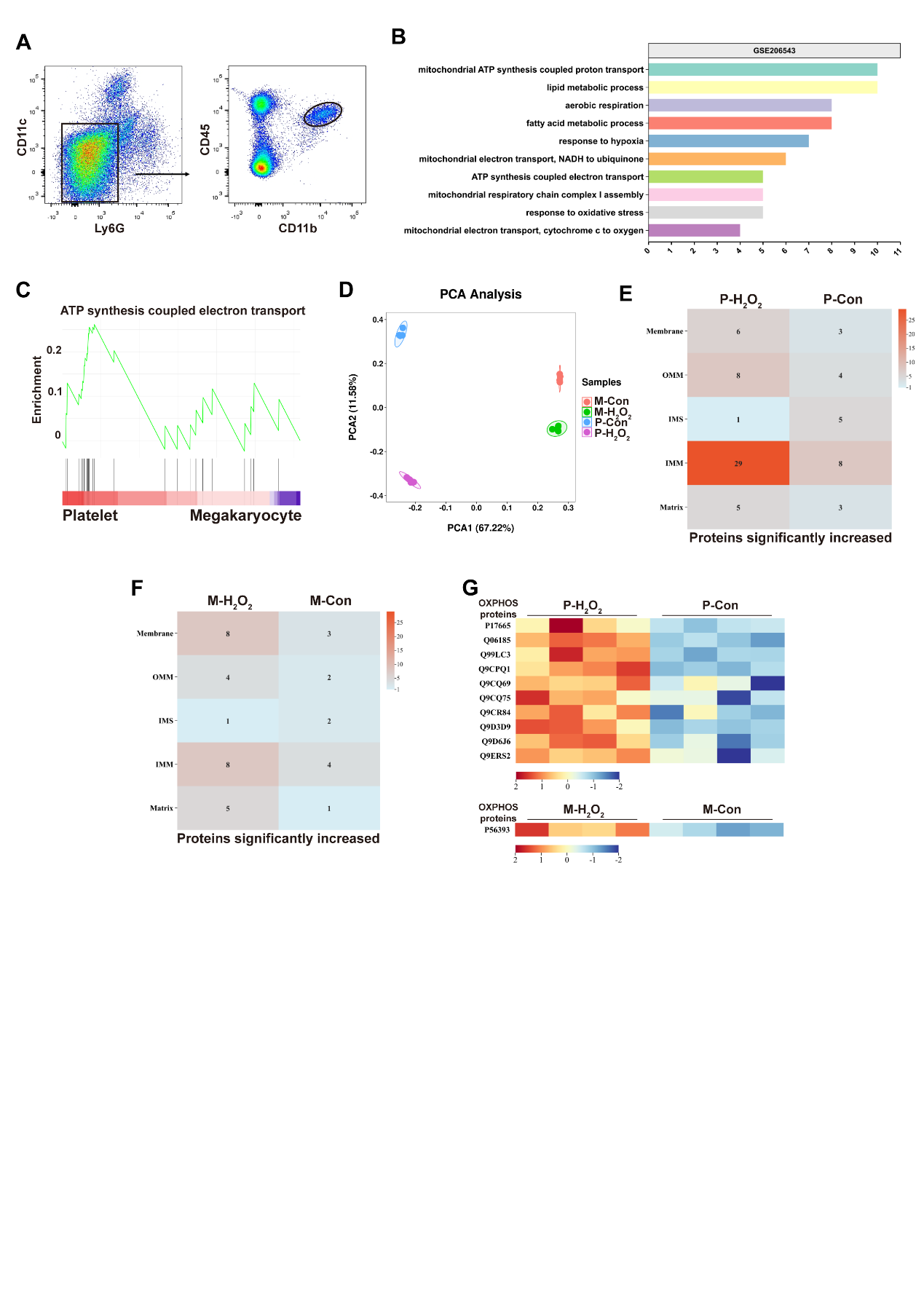


**Figure S1. Characterization of platelet and macrophage mitochondria.** **A)** Representative FACS density plots illustrating the cells sorted for ATP level analysis. The cells were gated as Ly6G^-^CD11c^-^CD11b^+^CD45^high^. **B)** Enriched GO pathways related to the significantly differentially expressed mitochondrial genes between megakaryocytes and platelets in the RNA-Seq dataset GSE206543. **C)** Gene set enrichment analysis was performed to compare ATP synthesis coupled with electron transport between megakaryocytes and platelets. **D)** PCA of mitochondrial proteins from the different samples. (n = 3). **E, F)** Heatmaps showing the mitochondrial localization of the proteins that were significantly increased in the indicated comparison. **G)** The expression of OXPHOS proteins significantly increased after H_2_O_2_ treatment in the platelet and BMMs groups. Annotations were obtained from MitoCarta 3.0.

**Figure S2**

**
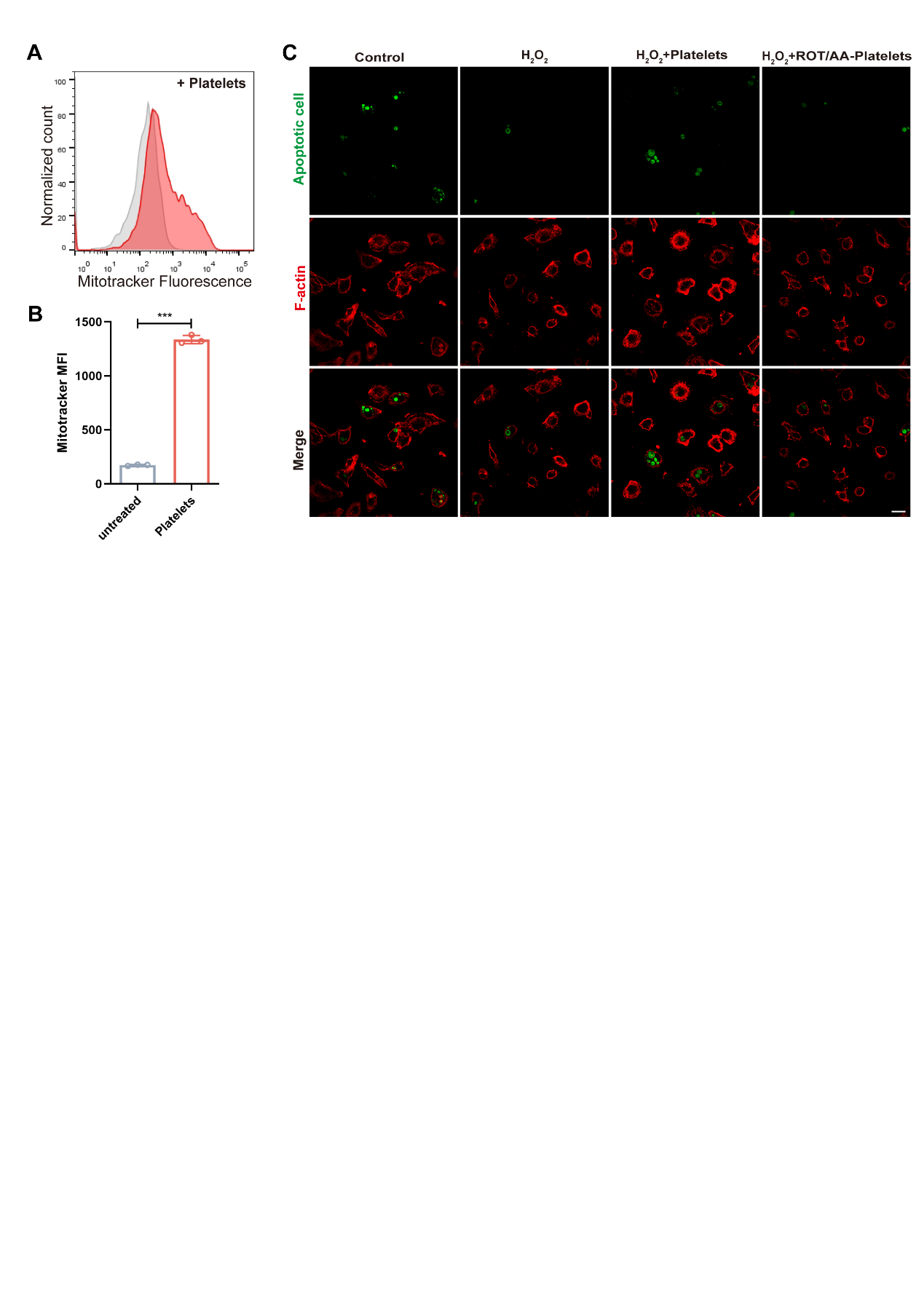
**

**Figure S2. Platelets stimulate macrophage efferocytosis. A)** Representative flow cytometry histogram of the internalization of MitoTracker Deep Red-labeled mitochondria from platelets in the lesion area. **B)** MFI of MitoTracker Deep Red (n = 3). **C)** Representative confocal microscopy analysis to evaluate efferocytosis by BMMs in vitro. Apoptotic Jurkat cells are in green; BMMs are in red. Scale bar, 20 µm. All data are depicted as means ± SD; ***P < 0.001.

**Figure S3**


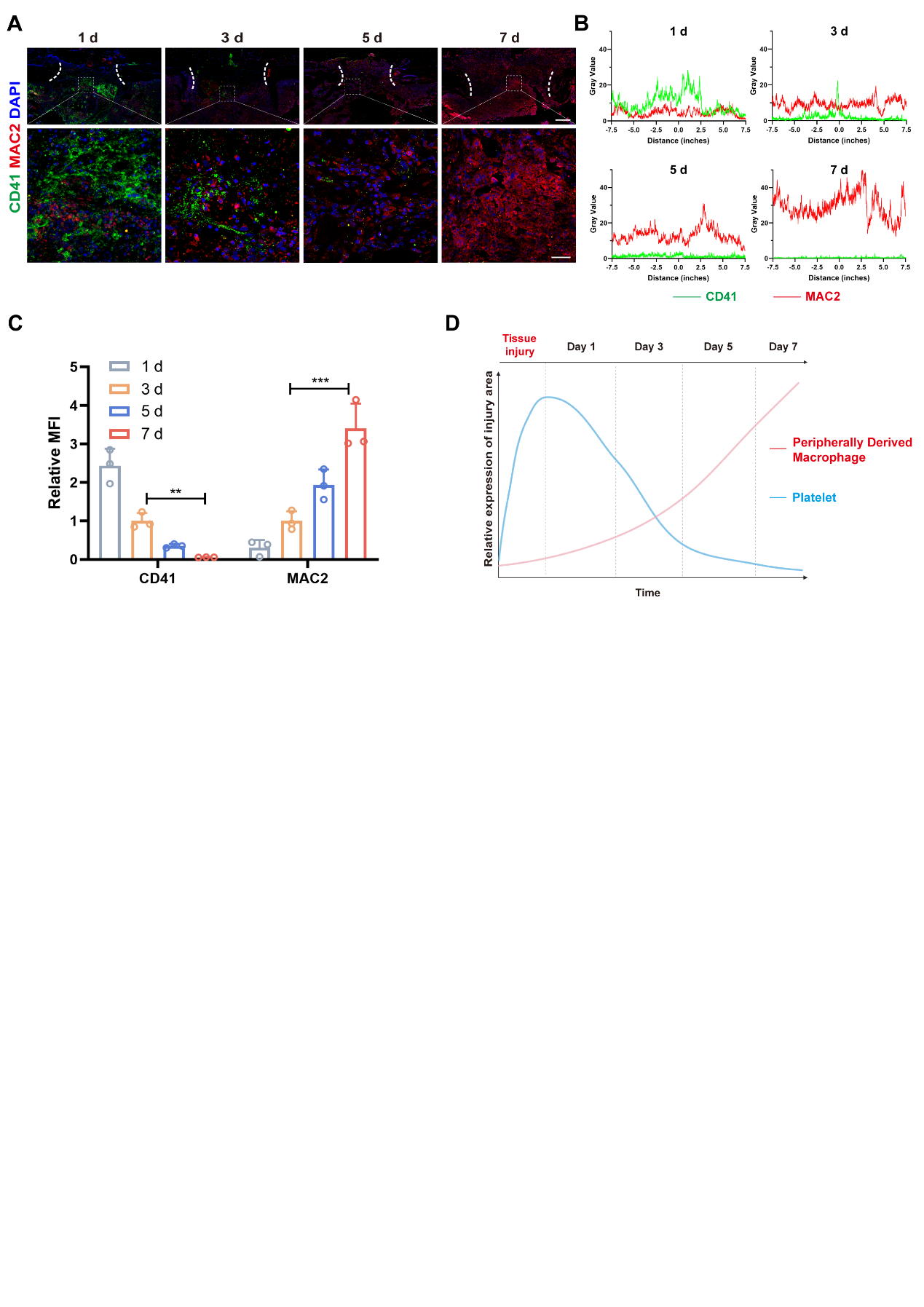


**Figure S3. Distribution of platelets and macrophages following tissue injury.** **A-C)** Immunofluorescence staining (**A**) and quantification (**B** and **C**) of CD41 (green) and MAC2 (red) in the femoral defect model (n = 3). Scale bar, 200 µm. Enlarged scale bar, 50 µm. **D)** Schematic diagram of the dynamic characterization of platelet- and peripherally derived macrophage infiltration after tissue injury. All data are depicted as means ± SD; **P < 0.01, or ***P < 0.001.

**Figure S4**

**
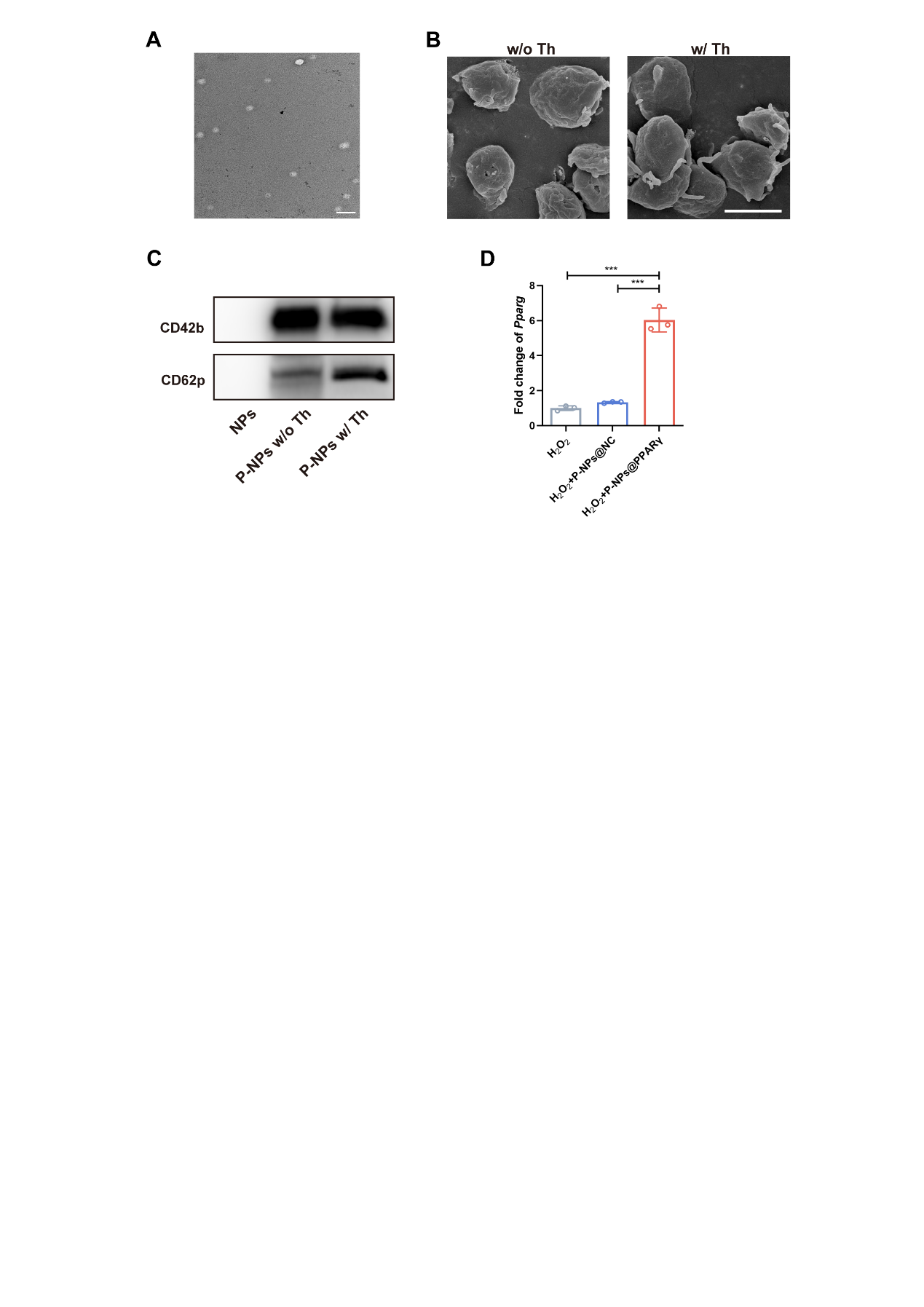
**

**Figure S4.** **Stress response and gene transfection of the NPs.** **A)** Transmission electron microscopy (TEM) image of the NPs. Scale bar, 200 nm. **B)** Representative SEM images of P-NPs@DNA with or without 0.5 U/ml Th treatment. **C)** WB images of platelet proteins in P-NPs with or without 0.5 U/ml Th treatment, where NPs were used as a control. **D)** Relative *Pparg* mRNA levels (n = 3). All data are depicted as means ± SD; **P < 0.01.

**Figure S5**


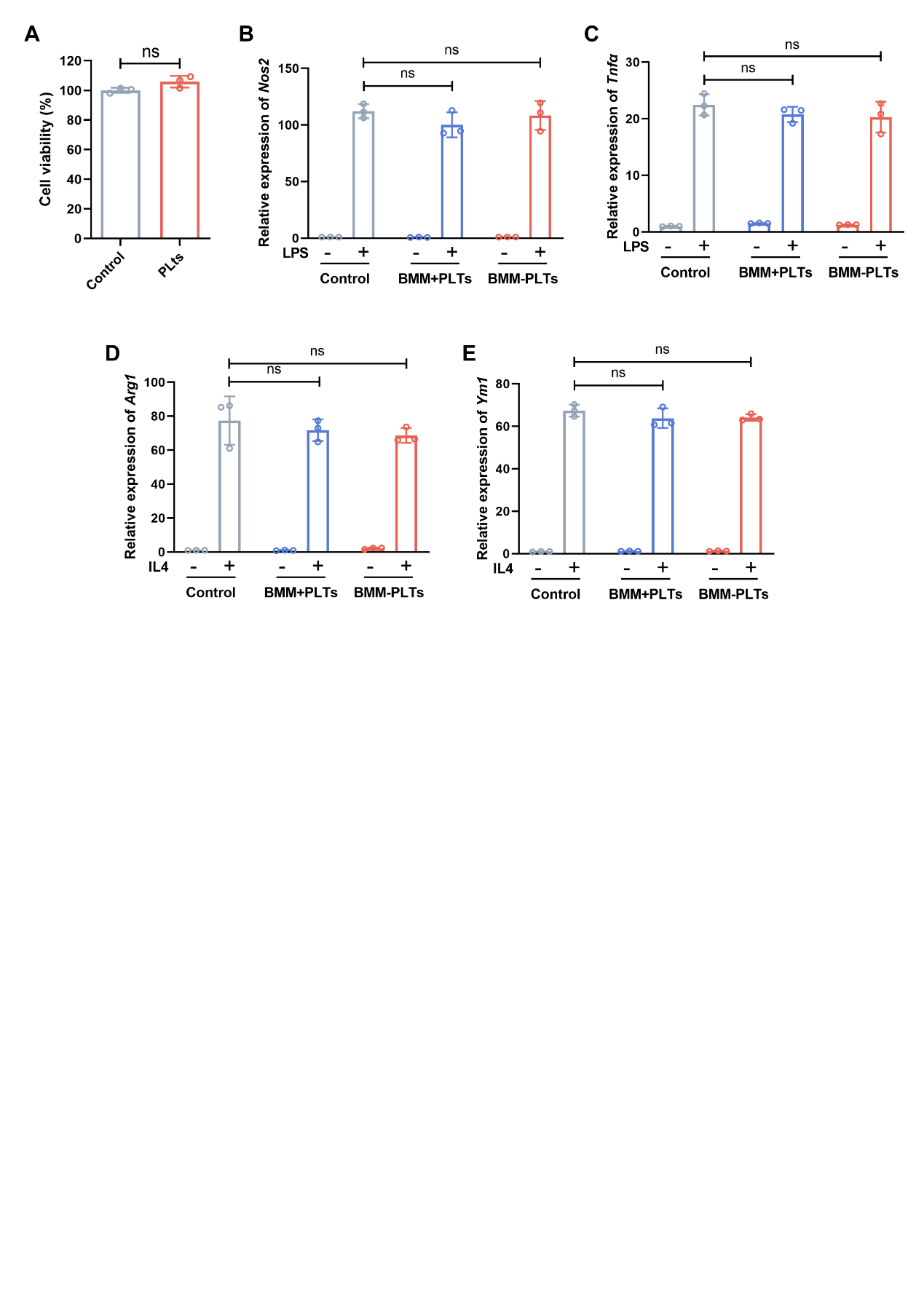


**Figure S5. Effect of platelets in macrophage function. A**) the cell viability of macrophages measured by CCK-8 with platelets treatment. **B**-**E**) The mRNA expression of *Nos2*, *Tnfα*, *Arg1* and *Ym1* under different treatment conditions with or without LPS/IL4 stimulation. All data are depicted as means ± SD; ns, not significant.

**Figure S6**

**
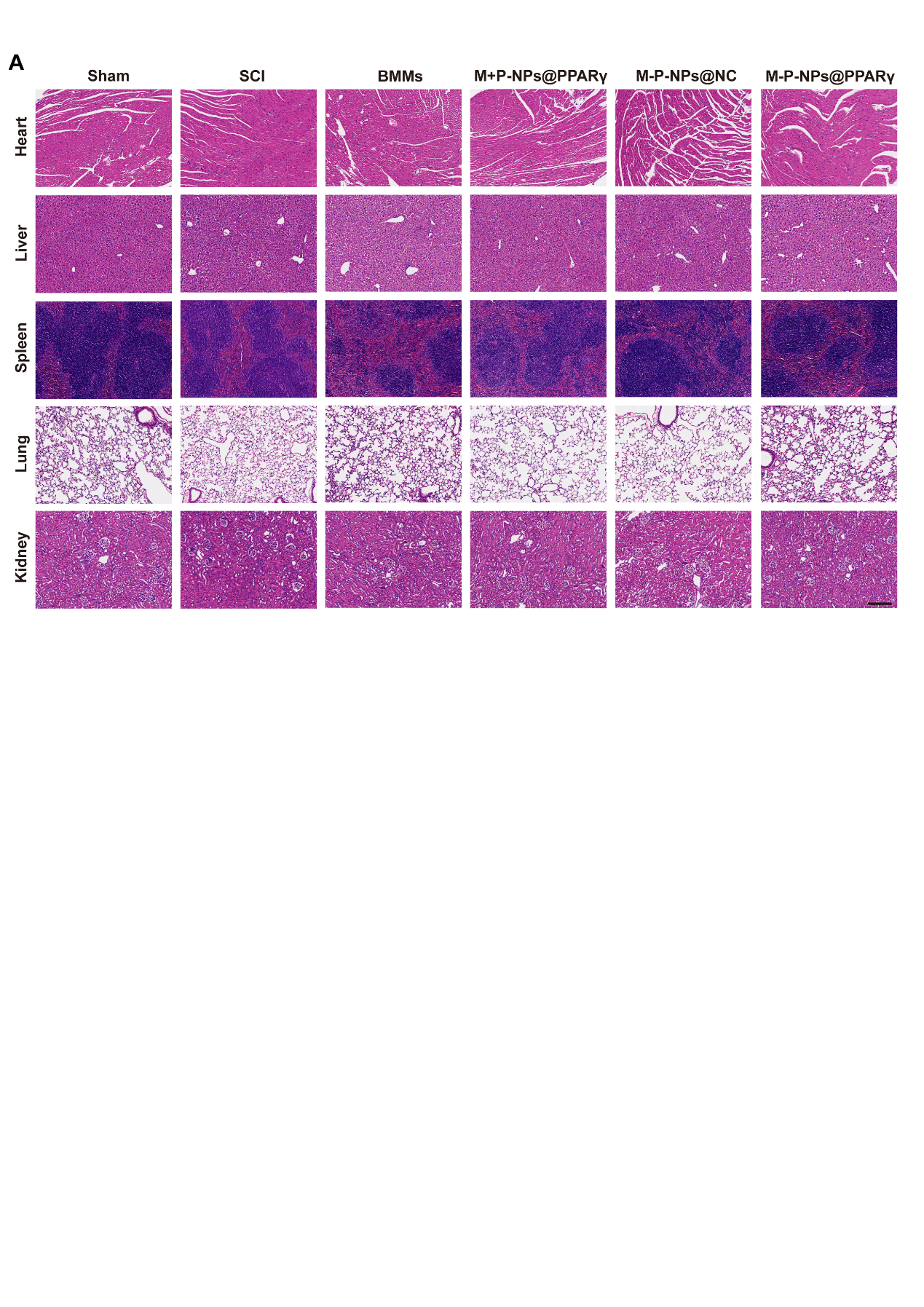
**

**Figure S6. In vivo biosafety of M-P-NPs@PPARγ.** **A)** H&E staining of structural integrity and inflammatory cell infiltration in the heart, liver, spleen, lung and kidney tissues of mice after M-P-NPs@PPARγ injection (scale bar, 200μm).
